# MXene-Ti_3_C_2_T_x_-Based Neuromorphic Computing: Physical Mechanisms, Performance Enhancement, and Cutting-Edge Computing

**DOI:** 10.1007/s40820-025-01787-0

**Published:** 2025-05-23

**Authors:** Kaiyang Wang, Shuhui Ren, Yunfang Jia, Xiaobing Yan, Lizhen Wang, Yubo Fan

**Affiliations:** 1 Medical Engineering & Engineering Medicine Innovation Center, Hangzhou International Innovation Institute, Beihang University, Hangzhou, 311115 People’s Republic of China; 2 Key Laboratory of Biomechanics and Mechanobiology (Beihang University), Ministry of Education, Beijing Advanced Innovation Center for Biomedical Engineering, School of Biological and Medical Engineering, Beihang University, Beijing, 100191 People’s Republic of China; 3 College of Electronic Information and Optical Engineering, Nankai University, Tianjin, 300071 People’s Republic of China; 4 Key Laboratory of Brain-Like Neuromorphic Devices Systems of Hebei Province College of Electron and Information Engineering, Jiaruiyuan Biochip Research Center of Hebei University, Hebei University, Baoding, 071002 People’s Republic of China

**Keywords:** Neuromorphic device, MXene-Ti_3_C_2_T_x_, Physical mechanisms, Performance improvement, Cutting-edge computing

## Abstract

Neuromorphic devices have shown great potential in simulating the function of biological neurons due to their efficient parallel information processing and low energy consumption. MXene-Ti_3_C_2_T_x_, an emerging two-dimensional material, stands out as an ideal candidate for fabricating neuromorphic devices. Its exceptional electrical performance and robust mechanical properties make it an ideal choice for this purpose. This review aims to uncover the advantages and properties of MXene-Ti_3_C_2_T_x_ in neuromorphic devices and to promote its further development. Firstly, we categorize several core physical mechanisms present in MXene-Ti_3_C_2_T_x_ neuromorphic devices and summarize in detail the reasons for their formation. Then, this work systematically summarizes and classifies advanced techniques for the three main optimization pathways of MXene-Ti_3_C_2_T_x_, such as doping engineering, interface engineering, and structural engineering. Significantly, this work highlights innovative applications of MXene-Ti_3_C_2_T_x_ neuromorphic devices in cutting-edge computing paradigms, particularly near-sensor computing and in-sensor computing. Finally, this review carefully compiles a table that integrates almost all research results involving MXene-Ti_3_C_2_T_x_ neuromorphic devices and discusses the challenges, development prospects, and feasibility of MXene-Ti_3_C_2_T_x_-based neuromorphic devices in practical applications, aiming to lay a solid theoretical foundation and provide technical support for further exploration and application of MXene-Ti_3_C_2_T_x_ in the field of neuromorphic devices.

## Highlights

- This review reveals the advantages of MXene-Ti_3_C_2_T_x_ for neuromorphic devices, classifies the core physical mechanisms, and outlines strategies to drive targeted optimization and future innovation.
- The review outlines three key engineering strategies: doping engineering, interfacial engineering, and structural engineering, while also providing comprehensive guidance for material and device improvement.
- MXene-Ti_3_C_2_T_x_-based devices demonstrate groundbreaking potential in next-generation computing, such as near-sensor computing and in-sensor computing, enabling faster and more energy-efficient data processing directly at the sensor level.

## Graphical abstract

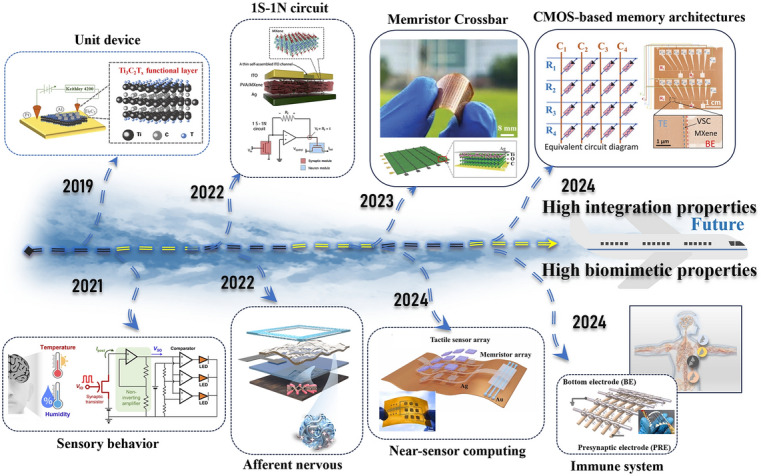

## Introduction

Artificial intelligence technologies have further enhanced the global computing infrastructure, with breakthroughs in high-performance computing and intelligent computing greatly improving the ability of computers to handle large-scale data and complex computing tasks [1–3]. However, the rapid development of artificial intelligence technology has increasingly demanded data intensification and resource miniaturization. The traditional separation architecture of perception, storage, and computing is gradually unable to meet the requirements of rapid processing of massive data and low-energy information transmission, becoming a bottleneck problem in the development of the artificial intelligence field [4, 5]. The neuromorphic system that integrates sensing, storage, and computing functions can perceive external signals while storing and computing in real-time, quickly, and efficiently simulating brain thinking, bringing new development opportunities to the field of artificial intelligence. This computing method often relies on emerging neuromorphic devices, which are electronic components that can mimic the function of biological neurons and synapses [6, 7]. For example, memristors can modulate their resistance value based on the accumulated charge or passed voltage, and synaptic transistors can modulate the channel’s conductivity by controlling the gate voltage [8, 9]. Traditional memristors and synaptic transistors have encountered challenges in terms of size reduction, energy consumption, and stability. Therefore, many researchers are dedicated to finding new electronic materials to enhance and improve the performance of neuromorphic devices.

**Fig. 1:**
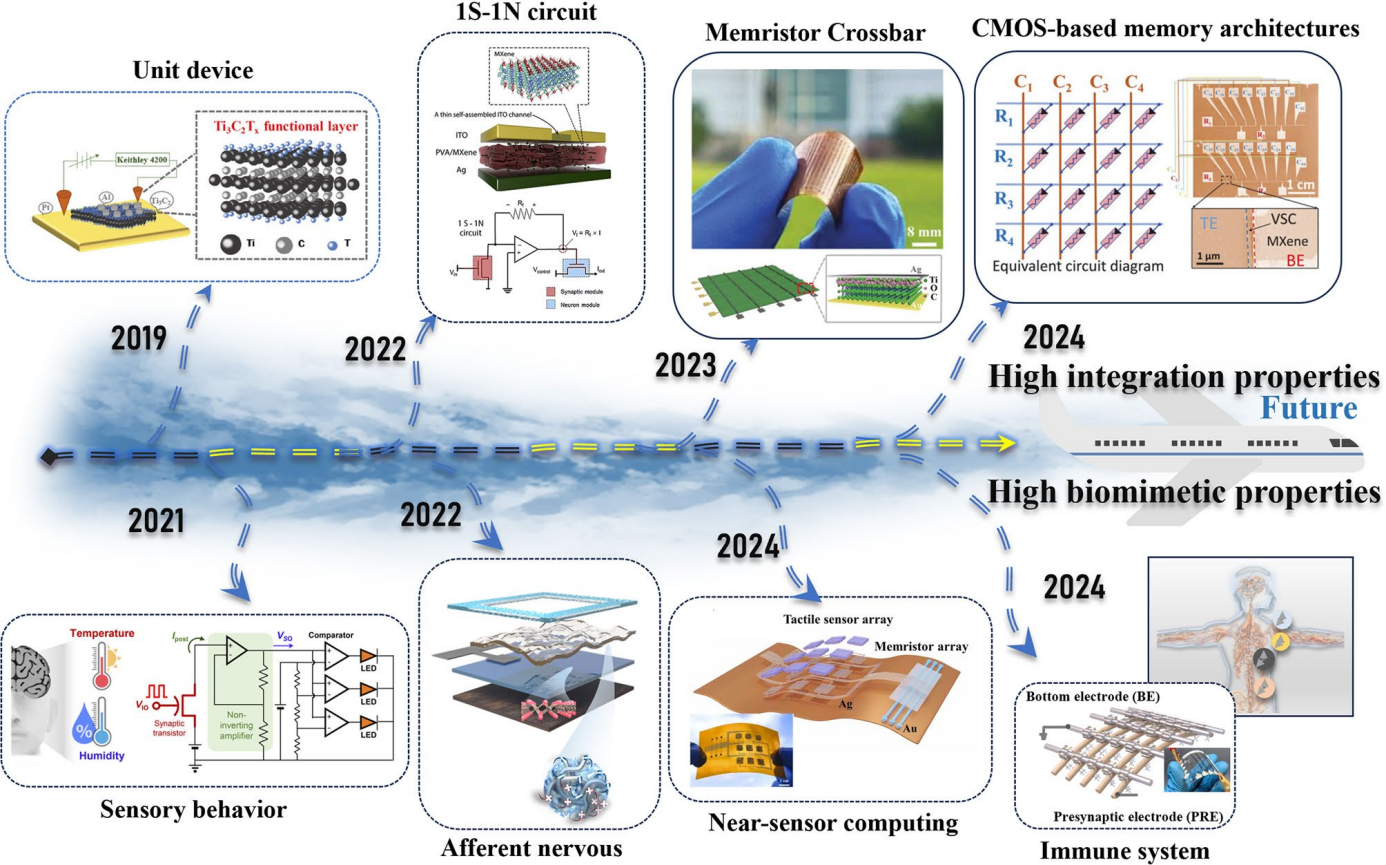
Historical development of MXene-Ti_3_C_2_T_x_-based neuromorphic device. Reproduced with permission [12]. Copyright 2019, WILEY‐VCH Verlag GmbH & Co. KGaA, Weinheim. Reproduced with permission [28]. Copyright 2021, American Chemical Society. Reproduced with permission [26]. Copyright 2022, Elsevier Ltd. Reproduced with permission [23]. Copyright 2022, Elsevier Inc. Reproduced with permission [24]. Copyright 2023, Wiley–VCH GmbH. Reproduced with permission [31]. Copyright 2024, Elsevier Ltd. Reproduced with permission [25]. Copyright 2024, Wiley–VCH GmbH. Reproduced with permission [28]. Copyright 2024, Elsevier Ltd. [29]

Two-dimensional (2D) nanomaterials are single-layer or few-layer novel nanomaterials, which can be prepared by a variety of methods, such as chemical vapor deposition, mechanical exfoliation, and solution exfoliation [10, 11]. So far, many types of 2D materials have been discovered so far, for example, graphene, MXene [12], MoS_2_ [13], black phosphorus (BP) [14], and h-BN [15]. MXene, as an emerging class of layered two-dimensional nanomaterials, consists of transition metal carbides or carbon nitrides bonded in a specific manner [16, 17]. Its structural features can be expressed by the general chemical formula M_n+1_X_n_ (where n ranges from 1 to 3). In this formula, M represents a series of transition metal elements, including scandium, titanium, zirconium, vanadium, molybdenum, etc., which endow MXene with excellent metallic properties. X stands for carbon or nitrogen, introducing unique electronic structures and surface chemistry to MXene [18, 19]. In the MXene family, MXene-Ti_3_C_2_T_x_ has become a hot topic of current research due to its extremely high specific surface area. Meanwhile, MXene-Ti_3_C_2_T_x_ has a variety of functional groups on the surface, such as -OH, -F, and -O. The presence of these functional groups not only enriches the surface chemistry of the material but also provides a wide range of opportunities for functionalization and performance modulation [20, 21]. Compared to the limited surface chemical tunability of graphene, the lower intrinsic conductivity of MoS_2_, and the environmental instability of black phosphorus, MXene exhibits a unique combination of advantages: its high surface area ratio, excellent electrical conductivity, optically sensitive properties, high mechanical strength, and other notable characteristics. These advantages show great potential in enhancing the performance and expanding the functionality of the memristor. For instance, neuromorphic devices can easily achieve saturation of output current and a lower SET voltage due to the ultra-thin thickness and native oxidation of MXene, thereby overcoming the high power consumption bottleneck inherent in traditional computing architectures [22]. Due to its unique properties, MXene-Ti_3_C_2_T_x_ exhibits promising applications in numerous fields, including energy, electronics, environment, biomedicine, and has gradually become a hotspot and focal point of research across many disciplines. It is worth mentioning that since the reporting of neuromorphic devices based on the MXene-Ti_3_C_2_T_x_ material around 2019 [12], as shown in Fig. 1, their demonstrated excellent electrical performance and high biomimetic neurotransmission efficiency have garnered widespread attention and sparked a wave of enthusiastic research within the scientific community. This breakthrough discovery brings new light to the field of 2D materials-based neuromorphic computing. Further, MXene-Ti_3_C_2_T_x_-based 1S−1N circuits [23], memristor cross-arrays [24], and CMOS-based memory structures not only pave the way for the realization of programmable neuromorphic chips with high precision and high efficiency, but also establish a solid foundation for a diverse range of applications of MXene-Ti_3_C_2_T_x_ in modern integrated circuits [25]. Meanwhile, the unique electron transport properties and high chemical sensitivity of MXene-Ti_3_C_2_T_x_ have demonstrated its high potential for application in the field of biomimetic neuromorphic electronics. For example, MXene-Ti_3_C_2_T_x_ neuromorphic devices have successively realized the construction of sensory nerves and afferent nerves [26–28], thus endowing them with more anthropomorphic features. Additionally, immune response simulation offers robust support in biomedical and other application scenarios that require high reliability and safety [29, 30]. In a word, these researchers seek to explore their potential applications in the fields of wearable devices, near-sensor computing, and in-sensor computing, with the ultimate goal of constructing neuromorphic systems that are smarter, more efficient, and capable of autonomous learning [31].

**Fig. 2:**
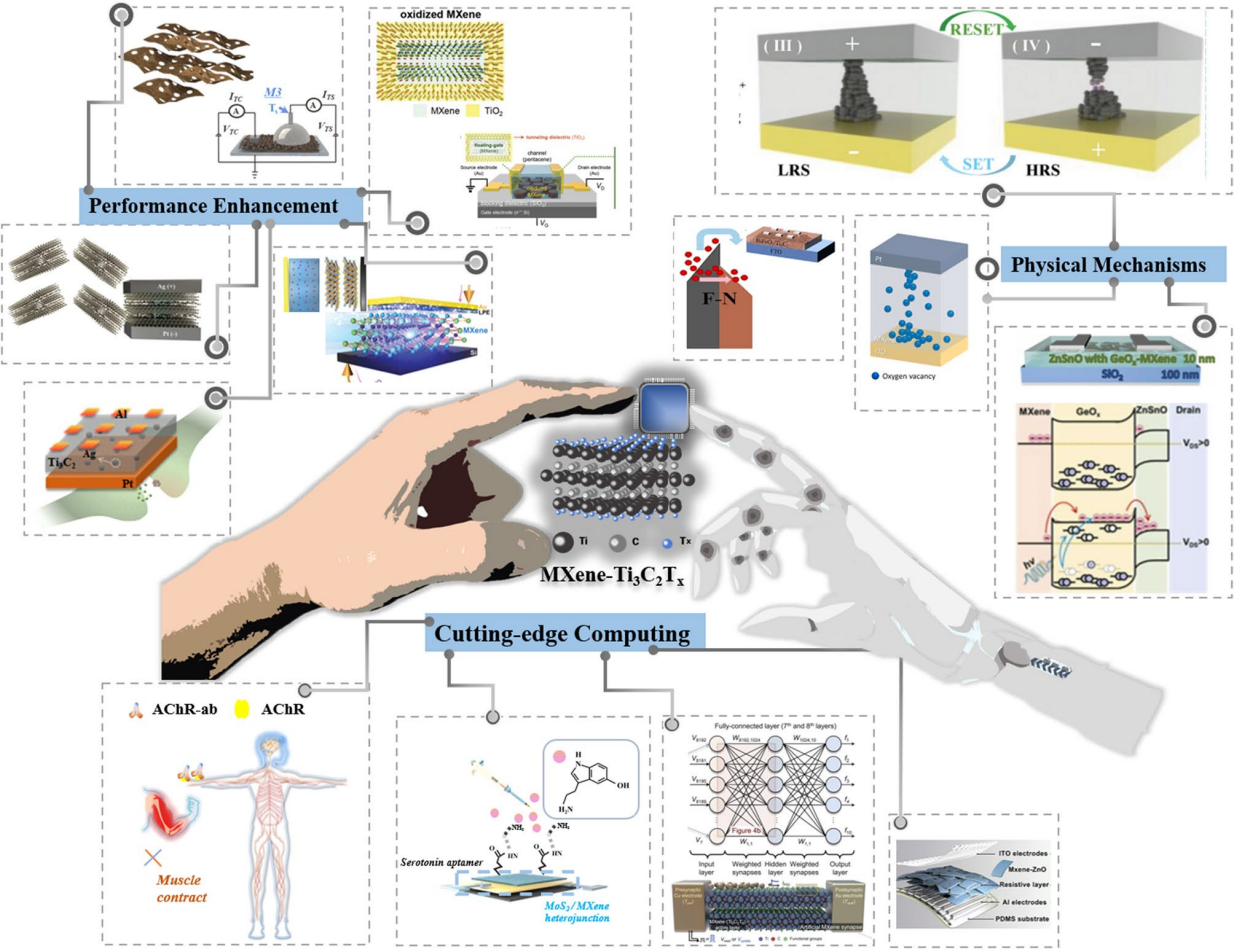
Classification of research routes for MXene-Ti_3_C_2_T_x_-based neuromorphic devices includes performance enhancement methods, exploration of physical mechanism and in-sensor computing. Reproduced with permission [30]. Copyright 2021, Wiley–VCH GmbH. Reproduced with permission [32]. Copyright 2020 Elsevier Ltd. Reproduced with permission [17]. Copyright 2020, Wiley–VCH GmbH. Reproduced with permission [37]. Copyright 2020, WILEY–VCH Verlag GmbH & Co. KGaA, Weinheim. Reproduced with permission [38]. Copyright 2023, Wiley–VCH GmbH. Reproduced with permission [39]. Copyright 2023, Elsevier Ltd. Reproduced with permission [40]. Copyright 2023, Elsevier Ltd. Reproduced with permission [41]. Copyright 2021, Wiley–VCH GmbH. Reproduced with permission [42]. Copyright 2021 Wiley–VCH GmbH

This review provides a comprehensive and timely summary of MXene-Ti_3_C_2_T_x_-based neuromorphic devices from three dimensions, which are performance enhancement, physical mechanism, and cutting-edge computing, as shown in Fig. 2. It aims to explore new ways to advance neuromorphic computing using MXene-Ti_3_C_2_T_x_ by summarizing its research progress and to further promote its practical applications in the fields of sense-storage-computing integration technology and artificial intelligence. This review firstly summarizes several physical mechanisms that have been reported in MXene-Ti_3_C_2_T_x_ neuromorphic devices, which provides a theoretical foundation for understanding how MXene-Ti_3_C_2_T_x_ works in neuromorphic computing [12, 32]. Second, the article also explores key strategies to enhance the performance of MXene-Ti_3_C_2_T_x_ neuromorphic devices, which are categorized into three aspects: doping engineering, interface engineering, and structural engineering [33, 34]. These efforts aim to build more efficient, reliable, and easy-to-integrate synaptic units for the underlying hardware of neuromorphic systems [35, 36]. Third, the article emphasizes cutting-edge computing modes based on MXene-Ti_3_C_2_T_x_ neuromorphic devices, including the innovative applications in near-sensor computing and in-sensor computing. It also elaborates the internal simulation strategy of the device using electrical signals, and the external simulation strategy using multimodal signals, and summarizes its exploratory applications in both integrated simulation computing and integrated hardware computing. Finally, this paper comprehensively summarizes almost all the research on MXene-Ti_3_C_2_T_x_-based neuromorphic devices, and at the same time insightfully points out the two core challenges faced by neuromorphic systems in the pursuit of highly integrated and bionic applications. This work aims to push the research on MXene-Ti_3_C_2_T_x_-based high-performance memristors to new heights and to provide a comprehensive and systematic framework for the future development of memory and neuromorphic computing systems based on novel 2D materials.

## Physical Mechanism

To overcome the barriers of neuromorphic devices in terms of reliability and durability, many researchers have explored the microphysical mechanism of charge transfer and switching processes with the help of the devices’ switching mechanism, aiming to achieve stable resistive switching state, long-term retention time, and high cycle characteristics [43–45]. Meanwhile, by investigating the working principle of the devices, researchers can achieve a more controllable switching process, which can further optimize switching speeds and energy consumption, and provide greater support for improving the performance of neuromorphic systems and realizing the integration of sensing, storage, and computing. The operating principles of neuromorphic devices can be classified into three categories: the electrochemical metallization (ECM) mechanism [46], valence change memory (VCM) mechanism [47], electron tunneling [12], and charge trapping.

### ECM

**Fig. 3:**
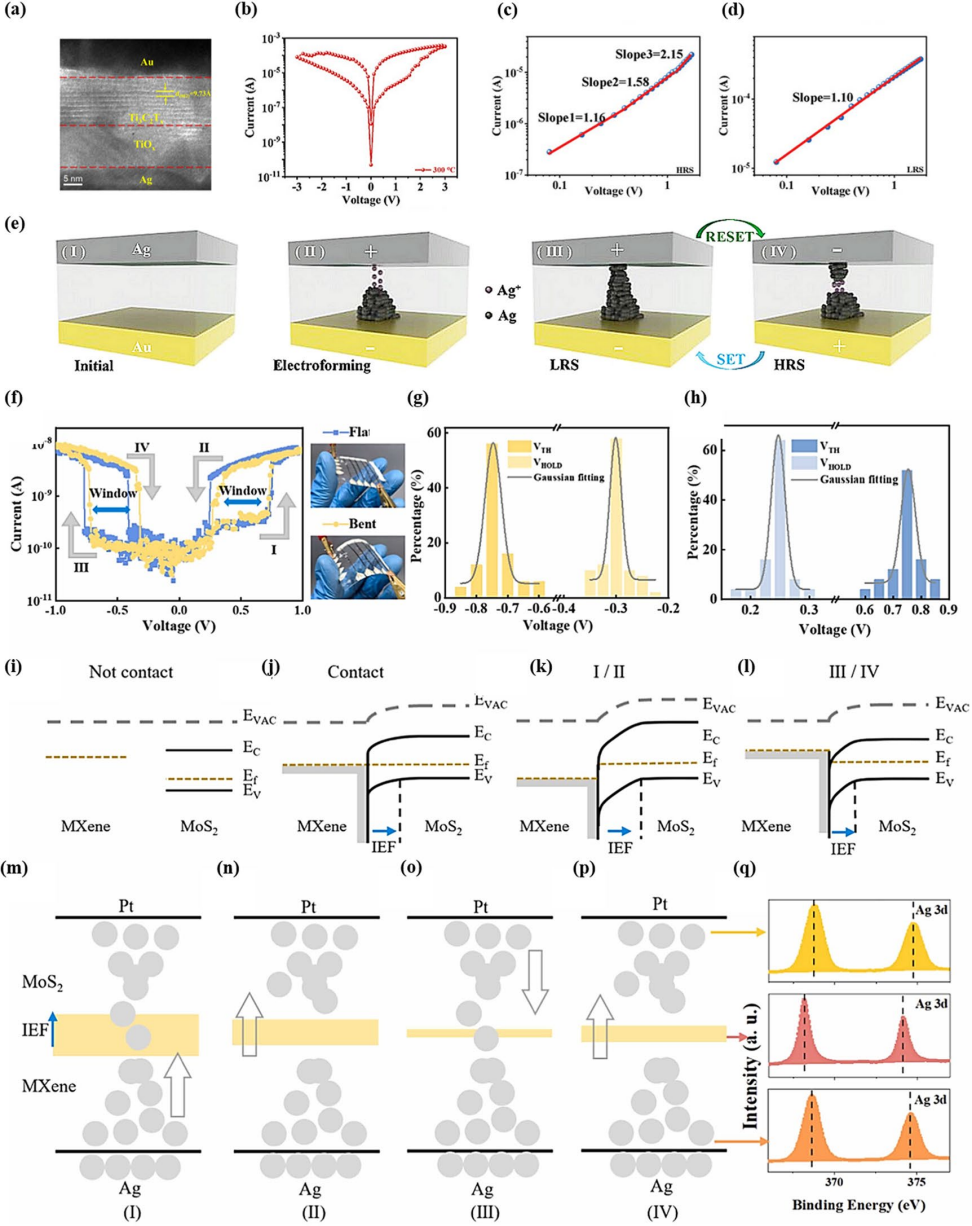
**a** HRTEM images of Ag/TiO_x_/Ti_3_C_2_T_x_/Au vertical heterostructure. **b** Typical I–V characteristic of the memristor. **c, d** I–V curves corresponding to HRS and LRS of the memristor, respectively, under the application of a positive electric field. **e** Schematic of RS mechanism. Reproduced with permission [24]. Copyright 2023, Wiley-VCH GmbH. **f** I-V characteristics of the device are shown when the PRE electrode voltages are swept between −1 and +1 V. The device is illustrated in the inserted photos when it is flat and softly bent, on the left and right sides, respectively. The forward and backward scanning processes at positive and negative voltages are labeled as I, II, III, and IV, respectively. **g,** **h** Turning voltages in processes I and III are named the threshold voltage (V_TH_), those in processes II and IV are named the hold voltage (V_HOLD_), and the hysteresis regions between them are termed the “window.” **i,** **j** Schematic diagrams of energy bands of the separated MXene and MoS_2_, and the bent energy bands of their heterojunction when they are contacted. **k,** **l** Energy bands of MXene/MoS_2_ heterojunction during processes of I/II and III/IV, respectively. **m-p** Schematic diagrams of the switching mechanisms of Ag conductive filaments at the interface of MXene/MoS_2_ in the measuring processes of I to IV **f**, respectively. **q** Ag 3*d* depth profile XPS spectra of the fibrous synapse. Reproduced with permission. Copyright 2024, Elsevier Ltd [29]

Neuromorphic devices dominated by ECM typically utilize active metals such as Ag, Cu, or Ti as top electrodes. In these devices, the formation and breakage of conductive filaments (CF) is the primary reason for the realization of resistive switching [48]. This process, which involves random ion migration, often leads to an uneven distribution of CFs within the functional layer, resulting in a diffuse distribution of device’s SET voltage and resistance states. This, in turn, limits their commercialization applications [49]. Therefore, improving the controllability of CFs is crucial for enhancing the performance aspects such as repeatability and endurance. Huang et al. reported a versatile and reliable solution processing method for 2D materials [24]. By utilizing spin-coating and a simple annealing process, compact and uniform TiO_x_/Ti_3_C_2_T_x_ heterostructure artificial synaptic arrays were successfully prepared at the wafer level, exhibiting high transmittance (≈90%) and excellent oxidation resistance (> 30 days). Figure 3a displays the vertical structure HRTEM image of the Ag/TiO_x_/Ti_3_C_2_T_x_/Au device. In this image, the Ti_3_C_2_T_x_ layer demonstrated good crystallinity, exhibiting clear lattice stripes with 9.73 Å crystallite spacing, which is consistent with the 002 crystalline phase of Ti_3_C_2_T_x_ [50]. Figure 3b shows the typical I-V characteristics of the TiO_x_/Ti_3_C_2_T_x_-based heterojunction device, exhibiting a bipolar nonvolatile resistive switching behavior. To further investigate the RS mechanism of the device, the positive voltage portion of the I-V curves corresponding to the HRS and LRS are replotted in double logarithmic coordinates, as illustrated in Fig. 3c, d. For the HRS, the I-V curve can be segmented into three regions with different slopes, which can be interpreted using the space charge limiting conduction (SCLC) model [51]. The slope of the fitted I-V curve in the first linear region is 1.16, aligning with the ohmic conduction mechanism. As the voltage increases, more metal cations migrate to the bottom electrode and are reduced to metal particles, leading to CF formation and a subsequent increase in current. Ultimately, it is observed that the current is proportional to the square of the voltage, which can be explained by Child’s law. For the LRS, once the Ag-CF channel is formed, the slope of the fitted I-V curves for all regions is 1.10, attributed to the high conductivity of the metal, following Ohm’s law [52]. Figure 3e visualizes the ECM-based RS behavior. Initially, the device is in HRS state. During the turn-on process, Ag atoms in the top electrode are oxidized to Ag^+^, which migrates from the top Ag electrode to the bottom Au electrode with positive bias. Subsequently, Ag^+^ are reduced to Ag particles at the bottom electrode and accumulates to form CF channels, and the resistive state of the device changes from HRS to LRS. When a negative bias is applied to the top electrode, due to Joule heating generated at the LRS, the CF channels break at the thinnest point in the middle, which triggers the resistive state of the memristor to return to HRS. Zeng et al. successfully developed a novel artificial optoelectronic memristor (OM-AOM) based on oxidized MXene, with the device structure of Ag/MXene/SiO_2_/Si. This study revealed the unique working mechanism of MXene as a resistive layer material in the memristor. First, the larger interlayer spacing of the MXene material provides an ideal diffusion channel for ion transport, effectively promoting the directional migration of Ag^+^ in the resistive layer. Second, UV irradiation was found to have a significant modulation effect on the device performance. When the device is in the high-resistance state (HRS), UV irradiation effectively accelerates the redox reaction of Ag, leading to the rapid formation of Ag-CFs in the MXene layers. This work provides new material design ideas for the development of high-performance memristors with photo-modulated properties [53]. An ITO/MXene-Ti_3_C_2_T_x_/EGaIn structured memristor is reported by Thomas et al. The combination of I-V results and AFM-based current mapping images demonstrated that the silver CFs do not completely disappear after applying lower RESET or erase voltages, resulting in a negative differential resistance region. Thus, the presence of Ag-CFs after the RESET process allows the device to never return to the original device conductance level [54]. Further, Wang et al. proposed a fibrous MXene artificial synaptic array with a heterojunction-modulated conductive filament model, as depicted in Fig. 3f. This model achieves precise modulation of the switching process [29]. The I-V test results indicate that the Ag/MXene/MoS_2_/Pt device exhibits typical threshold switching characteristics, as shown in Fig. 3f. The V_TH_ and V_HOLD_ during negative (V_PRE_ < 0) and positive (V_PRE_ > 0) scans were counted from 100 cyclic scans of the device and are plotted in Fig. 3g, h. The Gaussian fitted lines reveal that during positive scanning, V_HOLD_ and V_TH_ are concentrated at 0.25 and 0.75 V, respectively, while during negative scanning they are concentrated at −0.3 and −0.75 V, respectively. To reveal the underlying physical mechanisms, the researchers refer to work related to the energy band arrangement of MXene, MoS_2_, and their heterojunctions. Due to the different work functions of MXene and MoS_2_, as illustrated in the energy bands diagram in Fig. 3i, electrons flow from MXene to MoS_2_ upon contact, forming an internal electric field (IEF) at their interface that is oriented from MXene to MoS_2_, as depicted in Fig. 3j. When positive and negative V_PRE_ are applied, the IEF is enhanced and weakened, respectively, as shown in Fig. 3k, l. In the process I (Fig. 3m), as V_PRE_ increases in the forward direction, the IEF is gradually enhanced, facilitating the migration of Ag ions from the Ag electrode to the Pt electrode. These Ag ions gradually accumulate at the Pt electrode to form Ag-CF. When the V_PRE_ reaches to V_TH_, Ag-CF is completely formed, and the device switches to “on” state. As shown in Fig. 3n, in process II, as V_PRE_ gradually decreases, Ag ion migration is weakened.

There are Gaussian statistical distributions of V_TH_ and V_HOLD_ at positive and negative scanning voltages. When V_PRE_ is lower than V_HOLD_, the external electric field is unable to push the Ag ions to migrate to the MXene side. However, the natural IEF at the heterojunction can still drive the Ag ions from the MXene/MoS_2_ interface to the MoS_2_ side, resulting in the fracture of Ag-CF. In process III (Fig. 3o), as V_PRE_ is negatively increased, the IEF is gradually weakened, and its ability to transfer Ag ions to MoS_2_ is suppressed. When V_PRE_ becomes negatively high enough, the movement of Ag ions may be reversed, reconnecting the broken Ag-CFs at the heterojunction. As shown in Fig. 3p, as V_PRE_ decreases from -V_TH_ to -V_HOLD_ in the reverse direction, the function of the IEF to push the Ag ions from the MXene/MoS_2_ interface to the MoS_2_ side is gradually restored, allowing the Ag-CF to be disconnected again. The researchers subsequently validated the switching of Ag-CF by IEF through XPS depth profiling, as evidenced by the negative shift of the Ag-characteristic peak at the MXene/MoS_2_ interface in Fig. 3q [55].

### VCM

**Fig. 4:**
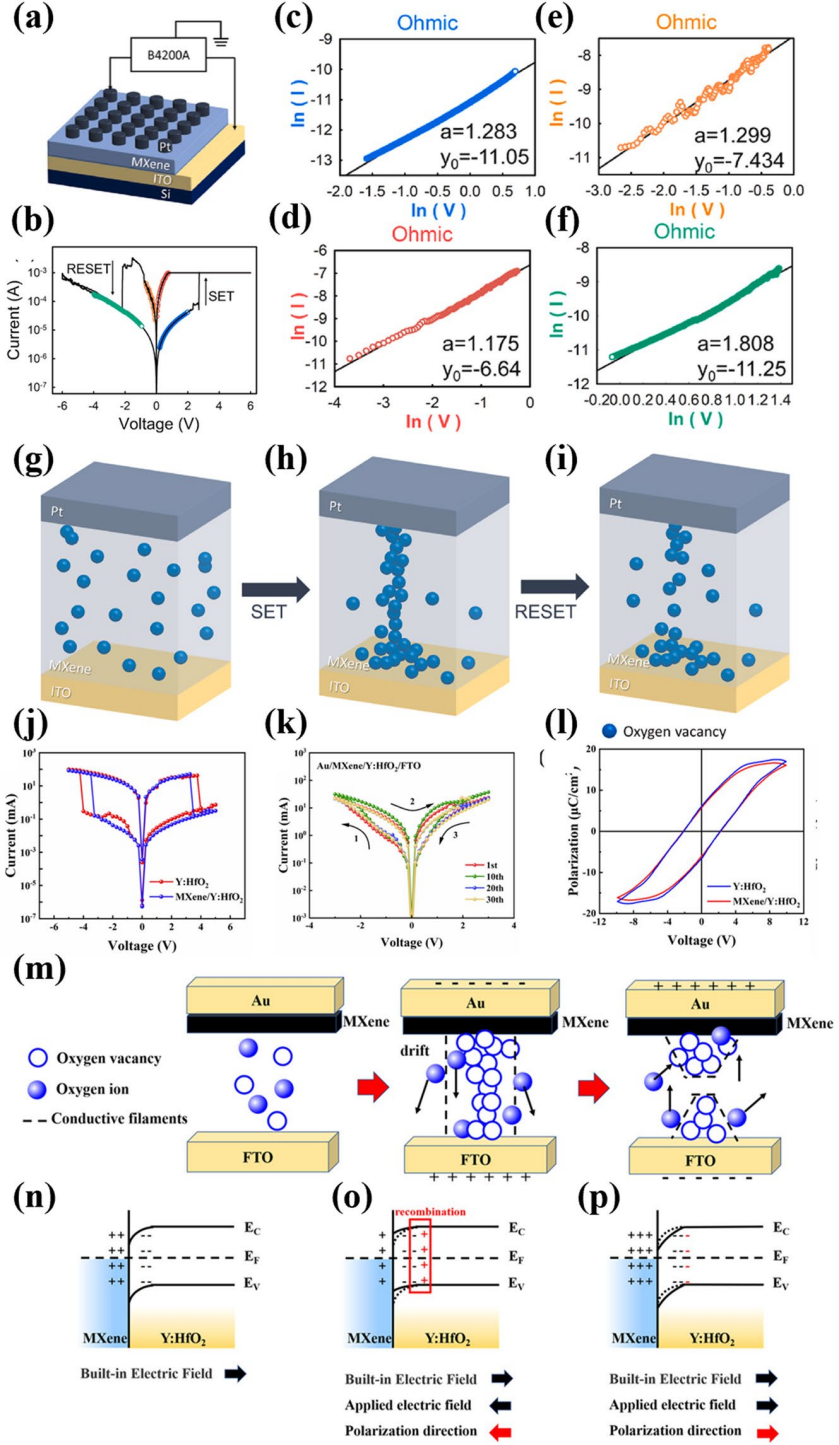
**a** Schematic illustration of the MXene-Ti_3_C_2_T_x_ memristor **b** Schematic analysis of HRS and LRS in the MXene-Ti_3_C_2_T_x_ memristor. **c** Ohmic relationship (slope was 1.283) at the HRS in region of 0.205 to 2.000 V in SET process. **d** Ohmic conduction (slope was 1.175) at the LRS in region of 0.765 to 0.025 V in SET process. **e** Ohmic relationship (slope was 1.299) at the LRS in region of −0.070 to −0.680 V in RESET process. **f** Ohmic conduction (slope was 1.808) at the HRS in region of −3.990 to −0.935 V in RESET process. Simple schematic illustration of the dynamic oxygen vacancy distribution in the MXene-Ti_3_C_2_T_x_ memristor: **g** original state, **h** on state, and **i** off state in resistance switching mechanism. Reproduced with permission [39]. Copyright 2023 Elsevier Ltd. **j** I-V curve of MXene/Y:HfO_2_ memristor with−5.0 to 5.0 V voltage. **k** I-V curve of MXene/Y:HfO_2_ memristor with−3.0 to 3.0 V voltage. **l** Ferroelectric hysteresis loop measured by the MXene/Y:HfO_2_ film and Y:HfO_2_ film. **m** Oxygen vacancy conductive filament model. **n–p** Schematic diagram of the energy band at the MXene/Y:HfO_2_ interface. Reproduced with permission. Copyright 2024 American Chemical Society [56] [53]

In the VCM, the formation of the conducting channel relies on the change in chemical valence of the active atoms within the functional layer, leading to the creation of a non-metallic CF composed of oxygen vacancies. For instance, Feng et al. reported an artificial synaptic device with a Pt top electrode, an oxidized MXene-Ti_3_C_2_T_x_ functional layer, and an ITO bottom electrode, as depicted in Fig. 4a [39]. The I-V curves of the device are presented in Fig. 4b. Refitting the I-V curves shown in Fig. 4c-f, the conductive mechanism of the MXene-Ti_3_C_2_T_x_ memristor was elucidated. In the case of HRS, the fitting results indicate that the current and voltage conform to Ohm’s law, exhibiting a linear relationship. This suggests that the number of free charge carriers generated by thermal excitation exceeds the number of injected carriers, and the trap sites are not fully occupied. In the HRS state, as depicted in Fig. 4c, f, the current is controlled by the ability of the negative electrode to inject charge. In the LRS state, a linear region is observed during SET at 0.765–0.025 V and RESET at −0.070–0.680 V, which can also be explained by Ohm’s law, indicating the formation of a conducting channel. The resistive switching mechanism of the memristor is then illustrated using VCM. When the device is in the initial state, the oxygen vacancies in the MXene-Ti_3_C_2_T_x_ film are distributed in a disordered manner, as shown in Fig. 4g. Upon applying a positive voltage stimulus, oxygen ions near the top electrode begin to migrate out of the resistive layer, generating oxygen vacancies (Vo^+^) within the film. These oxygen vacancies migrate toward the bottom electrode under the influence of a positive electric field, forming a non-metallic CF, as depicted in Fig. 4h. However, when a negative voltage is applied, the oxygen ions are captured by the top electrode, which results in the breaking of the CF and the subsequent switching of the device from LRS to HRS, as shown in Fig. 4i. Wang et al. reported a MXene-Ti_3_C_2_T_x_-based memristor structured as ITO/MXene-Ti_3_C_2_T_x_-ZnO/Al/PDMS. The authors conducted an in-depth profiling of the chemical state changes of the MXene-Ti_3_C_2_T_x_-ZnO film before and after SET operation using X-ray photoelectron spectroscopy (XPS). The results indicate that in the HRS, a significant amount of O^2−^ accumulates near the top Al electrode. Furthermore, when the device transitions to the LRS, the O^2−^ signal intensity near the Al electrode interface decreases, suggesting that O^2−^ migrates within the MXene-Ti_3_C_2_T_x_-ZnO and forms oxygen vacancy conductive filaments under the influence of an electric field [42]. Fang et al. prepared a ferroelectric memristor based on the Au/MXene/Y:HfO_2_/FTO structure, which exhibits digital-to-analog transition behavior [56]. As shown in Fig. 4j, the device demonstrates digital resistive switching behavior during continuous cyclic voltage scanning at ± 5 V. Figure 4k illustrates that when a cyclic voltage lower than the RESET voltage was applied, the resistance-change behavior of the device transitioned to an analog type. The device was then evaluated at room temperature using a ferroelectric analyzer, and the resulting hysteresis loops are presented in Fig. 4l at a load voltage of 10 V. The saturation and residual polarization strengths are similar for both the MXene/Y:HfO_2_ and Y:HfO_2_ films, suggesting that the addition of the MXene layer does not significantly alter the ferroelectric properties of the films. The VCM mechanism depicted in Fig. 4m is then explained through energy band theory. As shown in Fig. 4n-p, for the analog type, there exists an interfacial barrier between the HfO_2_ and the semimetallic MXene layer. In the HfO_2_ layer, a negative charge accumulates on the side adjacent to the MXene, generating a built-in electric field that points from the MXene toward the HfO_2_. When a negative bias voltage is applied to the top electrode, the direction of the applied electric field opposes the built-in electric field, leading to a reduction in the barrier height. Simultaneously, the ferroelectric polarization of the HfO_2_ film tends to shift upward, causing the positively polarized charges to migrate toward the MXene side. This process facilitates the recombination of electrons with the positively polarized charges at the MXene/HfO_2_ interface, thereby lowering the barrier height and transitioning the device to the LRS state. However, when a positive bias voltage is applied, the barrier height at the interface increases due to the combined effect of the applied electric field and the downward ferroelectric polarization, and the device transitions from LRS to HRS. For digital types, when a positive voltage is applied, the direction of ferroelectric polarization is aligned with the direction of the built-in electric field, inducing oxygen vacancy migration and thus promoting CF formation. On the contrary, when a negative voltage is applied, the direction of ferroelectric polarization is opposite to the direction of the built-in electric field, which helps to reduce the formed CF. Desai et al. prepared an organic–inorganic hybridized cellulose-Ti_3_C_2_T_x_ MXene composite hydrogel (CMCH) and then employed this material to construct Ag/CMCH/FTO structured memristors. Their study further revealed the physical mechanism underlying the cooperative interaction between ECM and VCM operating modes. The physical mechanism underlying the cooperation between ECM and VCM is revealed. Under forward bias, cations such as VO^2^⁺ and Ag⁺ in the CMCH layer migrate toward the FTO substrate, while anions (O^2^⁻) accumulate toward the Ag electrode. When the ion migration reaches a dynamic equilibrium, the accumulation of interfacial charges leads to a reduction in the effective dielectric layer thickness, causing the device to switch to the LRS. After reversing the bias polarity, some positive and negative ions at the interface recombine, resulting in an increase in the effective dielectric layer thickness and prompting the device to switch back to the HRS.

It is worth noting that when higher voltages are applied, the massive aggregation of Ag⁺ and VO^2^⁺ ions forms thicker conductive filaments. This process encompasses both the metal filament formation typical of the ECM mechanism and the oxygen vacancy migration characteristic of the VCM mechanism, ultimately demonstrating the cooperative nature of the two mechanisms [57].

### Electron Tunneling

**Fig. 5:**
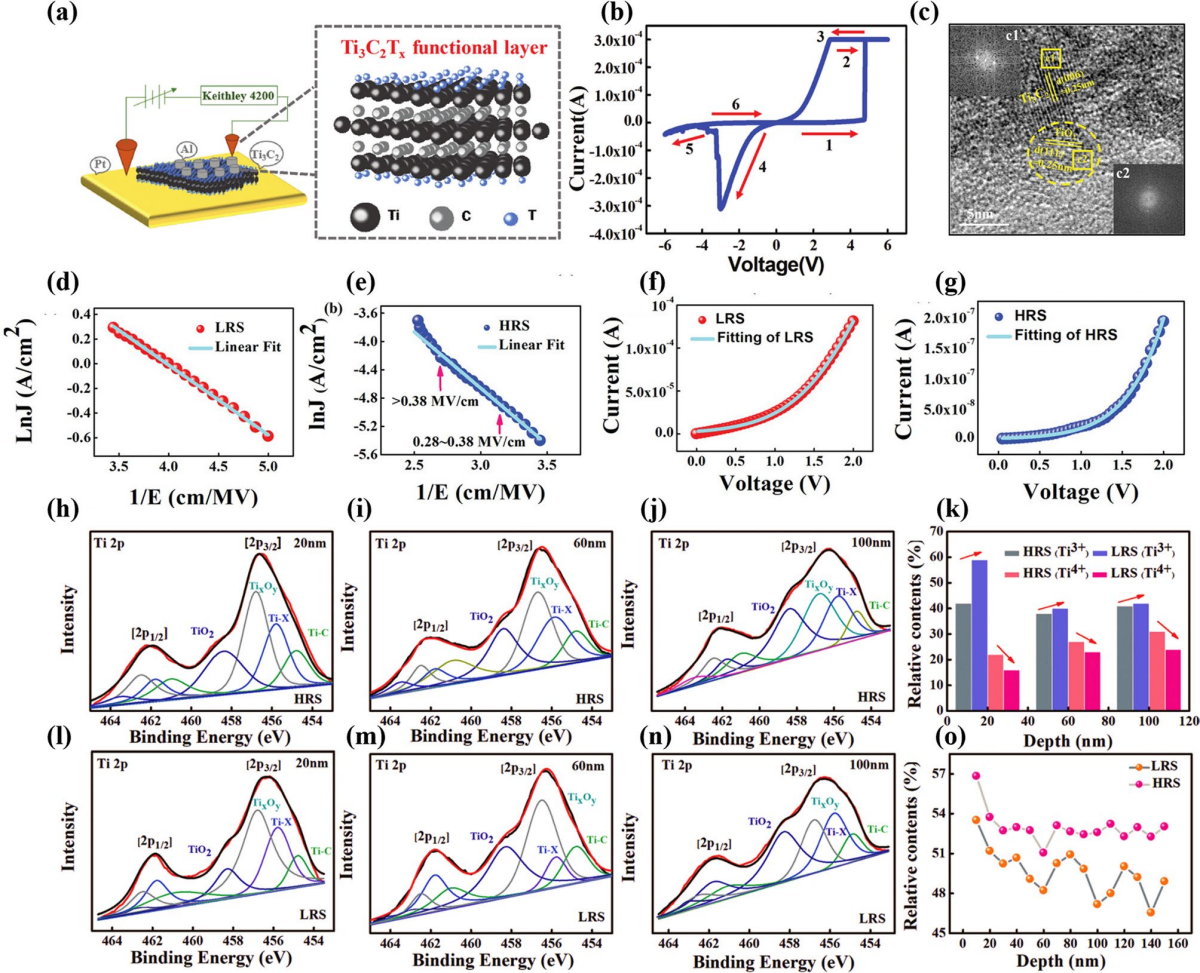
**a** Schematic structure of the Al/Ti_3_C_2_T_x_/Pt device and Ti_3_C_2_T_x_ atomic structure (T_x_ stands for surface termination). **b** Typical I-V curves of the device. **c** Part of the titanium carbide is oxidized to titanium oxide in the atmosphere. **d, e** Linear fittings with ln**j** ∝ 1/E for HRS and LRS indicate the TAT mechanism. **f, g** HRS and LRS of the I–V curve are fitted by Eqs. (1)–(4). XPS depth analysis of Ti_3_C_2_T_x_ flakes in the initial state and low-resistance state. **h, i** 20 nm. **l**, **m** 60 nm. **j, n** 100 nm. **k** Relative contents of Ti^3+^ and Ti^4+^ at different depths. **o** Oxygen content of HRS and LRS at different depths in the flakes. Reproduced with permission [12]. Copyright 2019, WILEY‐VCH Verlag GmbH & Co. KGaA, Weinheim

The electron tunneling mechanism is categorized into three cases: (1) When the functional layer is thin, the energy of the electrons exceeds the potential barrier, thus overcoming the barrier and tunneling to the other side, which is direct tunneling (DT) [58]. (2) When the functional layer is thick, the electrons cross the barrier to reach the other side, where the electrons undergo a longer distance and discontinuous tunneling process, which is called Fowler–Nordheim tunneling (FNT) [59]. (3) When there is a defect in the functional layer, the electron crosses the barrier with the assistance of a trap energy level, which is known as a trap-assisted tunneling mechanism (TAT) [60]. In this mechanism, introducing trap energy levels into the energy band structure can effectively enhance the tunneling probability of electrons, thereby generating a higher tunneling current. Furthermore, the density of traps exerts a modulating influence on the switching process of such memristors, and trap-assisted tunneling plays a pivotal role in the overall conductive process within the dielectric layer, particularly when the trap density is high. In neuromorphic devices, the three types of electrons tunneling effects occur alternately in response to changes in the functional layer material and applied voltage. As shown in Fig. 5a, a memristor with Al/Ti_3_C_2_T_x_/Pt structure was prepared by yan et al. [12]. The memristor exhibits a clear bipolar resistive switching behavior, as illustrated in Fig. 5b, and the current switching ratio exceeds two orders of magnitude, indicating that the device can fulfill the basic storage function. Subsequently, the physical mechanisms underlying the device’s switching process were further investigated. Based on the TEM images presented in Fig. 5c, it can be observed that there are Ti atomic vacancies and partially oxidized regions within the Ti_3_C_2_T_x_ nanosheets. Subsequently, the voltage–current curves of the devices were fitted according to the trap-assisted attempted penetration model, as depicted in Fig. 5d - g. The I-V curves for the low-resistance state (LRS) and high-resistance state (HRS) were fitted using the linear function lnj ∝ 1/E for the range from 0.2 to 0.38 MV cm^−1^ in Fig. 5d, e, where the conductance is dominated by a high electric field (> 0.38 MV cm^−1^). Two key parameters, the tunneling distance d and the trap energy *Et*, are subsequently fitted in Fig. 5f, g using Eqs. (1)–(4) [61, 62]. The tunneling current is modeled as [63]:

1 $$I=N\cdot q\cdot v$$

where *N* is the number of traps and *v* is the transition rate. The equation of v is as follows

2 $$v={v}_{0}\cdot f\cdot P$$

where *v*_0_ is the frequency factor. The equations for the Fermi–Dirac distribution f and transmission probability *P* are as follows:

3 $$f=l\setminus \left[1+\text{exp}\left(\frac{{E}_{b}-{E}_{t}+F\cdot d}{kT}\right)\right]$$

4 $$P=\text{exp}\left\{-\frac{4}{3\hbar qF}\sqrt{2m}\right\}\left[{E}_{t}^{3/2}-{\left({E}_{t}-F\cdot d\right)}^{3/2}\right]$$

where *E*_*b*_ is the barrier height, *k* is Boltzmann’s constant, *T* is the temperature, *ħ* is Planck’s constant, *q* is the electronic charge quantity, *F* is the electric field intensity, *Et* is the defect-trap energy below the CB, and *d* is the distance between the interface of Pt-Ti_3_C_2_T_x_ and the nearest trap site.

Figure 5f, g shows the I-V curve fitting results obtained by adjusting d and Et, assuming no change in *N*. After electroforming, *Et* slightly increases to 0.131 eV in HRS and d increases to 0.12 nm in LRS. After electroforming, in the HRS, *Et* slightly increases to 0.131 eV, while d increases to 0.12 nm. In contrast, in the LRS, *Et* is 0.105 eV and d is 0.08 nm. The results indicate that the HRS has a higher trap energy level (*Et*) and trap spacing *d*. Therefore, the lower trap energy and smaller trap spacing in the LRS are favorable for carrier transport, which is consistent with the typical trap-assisted tunneling (TAT) model. Consequently, the researchers hypothesized that the Al/Ti_3_C_2_T_x_/Pt memristor operates based on a TAT mechanism mediated by both titanium vacancies and oxygen vacancies. In order to confirm this result, the entire functional layer region under the A1 electrode was analyzed for elemental valence changes and elemental content in the high-resistance and low-resistance states, respectively. Figure 5h-j shows the XPS depth profiles at 20, 60, and 100 nm inside the functional layer of the device in the HRS. Figure 5l-n shows the XPS depth profiles at 20, 60, and 100 nm within the functional layer of the device in the LRS. The contents of Ti^3^⁺ and Ti^4^⁺ before and after switching are counted, as shown in Fig. 5k. It can be observed that Ti^4^⁺ decreases, while Ti^3^⁺ increases during the transition of the device from the HRS to the LRS, indicating an increase in oxygen vacancies. Figure 5o displays the change in oxygen content from the Al electrode to the Pt bottom electrode. It can be seen that the oxygen content decreases in the LRS, which corresponds to the increase in oxygen vacancies. Therefore, Ti vacancies and oxygen vacancies generated by Joule heating can serve as traps to assist carrier hopping during the resistive switching process of the device, facilitating the transition from a high-resistive state to a low-resistive state.

**Fig. 6:**
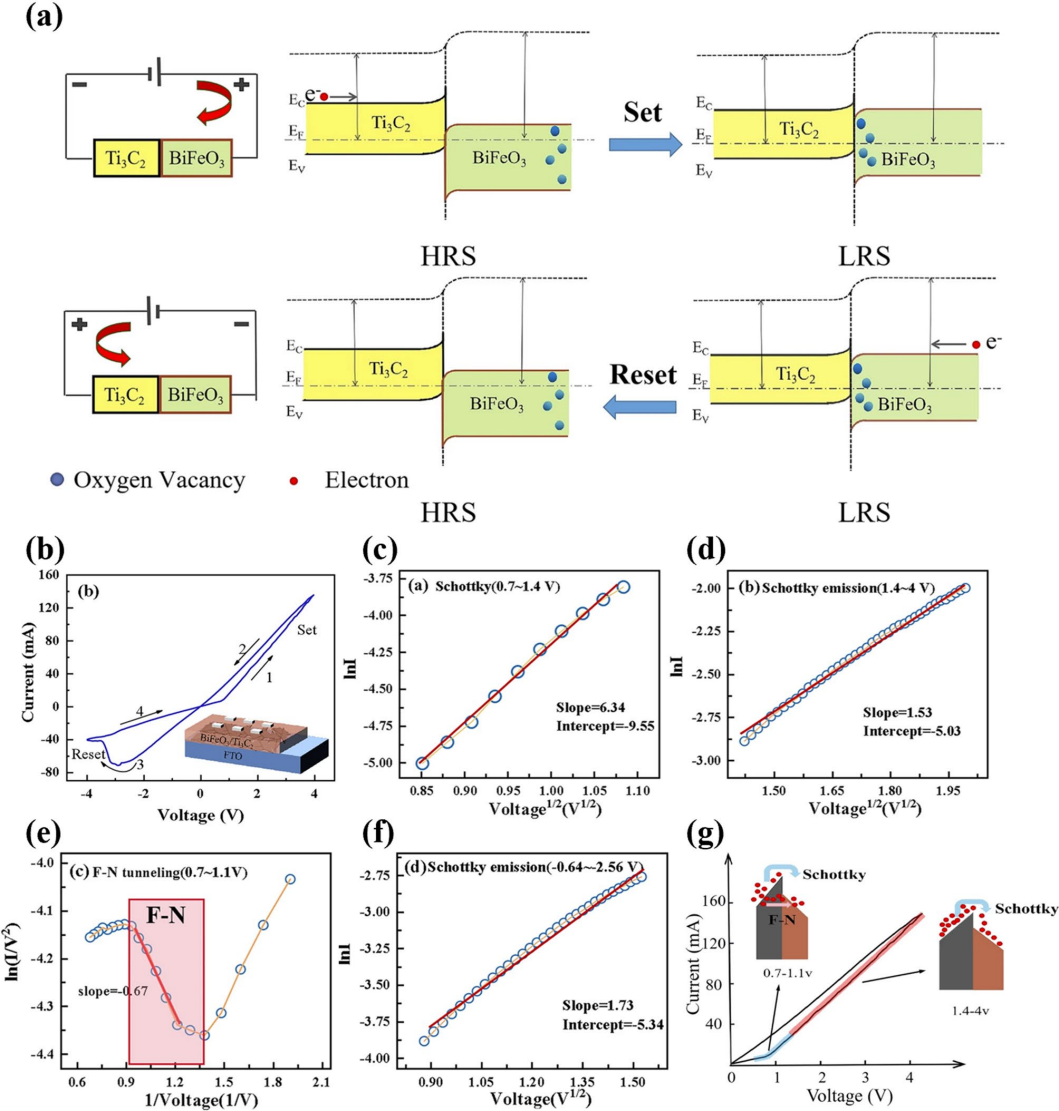
**a** Evolution of the band structures of the BiFeO_3_/Ti_3_C_2_T_x_ heterojunction under different electrical biases during the SET and RESET processes **b** Ag/BiFeO_3_/Ti_3_C_2_T_x_/FTO memristor; inset a schematic of Ag/BiFeO_3_/Ti_3_C_2_T_x_/FTO memristor. **c** Modeled Schottky emission curves for the devices of 0.7–1.4 V and **d** 1.4–4 V. **e** Modeled F-N tunneling curves for devices of 0.7–1.1 V. **f** Modeled Schottky emission curves for the device of—0.4 ~ − 2.56 V. **g** Schematic of the barrier change of the Ag/BiFeO_3_/Ti_3_C_2_T_x_/FTO memristor under the gradual increase in the forward bias voltage. Reproduced with permission [63]. Copyright 2023, The Minerals, Metals & Materials Society [65]

Sattar et al. reported a ferroelectric memristor with Ti_3_C_2_T_x_/HT-Ti_3_C_2_T_x_/BFO/Ti_3_C_2_T_x_ structure, which was tested and found to exhibit negative differential resistance effect (NDR) during turn-on [64]. The authors then analyzed the cause of NDR generation. When an external bias voltage is applied, the movable iron ions and free electrons in the active BFO layer jointly participate in the switching of the device. As a result, the device current increases and transitions from the HRS to the LRS state, exhibiting a linear ohmic characteristic. With further increase in voltage, cations accumulate at the cathode interface. When the voltage exceeds the threshold voltage, ion migration stops, and the current decreases sharply, leaving only the electrons to participate in conductivity. Consequently, the device is in the NDR low-conductance state and remains there as the voltage decreases. A photo-modulated memristor based on BiFeO_3_/Ti_3_C_2_T_x_ heterojunction has been reported by Qin et al. [65]. The resistance switching of this metal/heterojunction/metal structure memristor depends on the energy band modulation resulting from the coexistence of Ti_3_C_2_T_x_ and BiFeO_3_. The changes in the heterojunction bands during RESET and SET operations are shown in Fig. 6a. Due to the different Fermi energy levels and band gaps of the two materials, a potential barrier is formed at the interface. In the initial state, the device exhibits HRS. As the forward bias voltage increases, oxygen vacancies (blue spheres) gradually migrate to the interface and accumulate, causing the bending of the Fermi energy levels and leading to a decrease in the barrier height. When the voltage is gradually increased to the threshold voltage, the barrier height of the BiFeO_3_/Ti_3_C_2_T_x_ heterojunction decreases to its minimum, and the electrons on the heterojunction increase dramatically, causing the device transition from HRS to LRS.

When the device is under negative bias, the accumulated oxygen vacancies at the interface of the heterojunction gradually move back to the edges of the BiFeO_3_ energy bands, leading to the gradual restoration of the energy band structure of the BiFeO_3_/Ti_3_C_2_T_x_ heterojunction to its initial state. Based on this energy band theory, the memristor with an Ag/BiFeO_3_/Ti_3_C_2_T_x_/FTO structure exhibits a bipolar resistive switching characteristic, as shown in Fig. 6b. The conductive mechanism of the device was then further analyzed. The linear fit of Fig. 6c shows the dominance of the Schottky emission model for the device at 0.7–1.4 V, demonstrated by the fitting results of LnI ~ V^1/2^ in Fig. 6d. Figure 6e shows the F-N tunneling fitting results for the device at 0.7–1.1 V and indicates the presence of F-N tunneling in this region. The other voltage regions exist solely in the form of Schottky emission, as shown in Fig. 6f. These results indicate that the conductive mechanism of the device is primarily based on Schottky emission and F-N tunneling. Figure 6g visualizes the schematic of the device barrier change when forward bias is increased. At 0.7 to 1.4 V, Schottky emission and F-N tunneling coexist. F-N tunneling dominates due to the high potential barrier. As the voltage exceeds 1.4 V, the potential barrier becomes thicker, and electrons are unable to cross it, so the mechanism shifts to Schottky emission.

In addition, there is a positive correlation between electron tunneling and the weak formation of Ag-CFs. Ren et al. reported fibrous artificial synapses structured as PLA/Ag/MXene/Pt, and they demonstrated the physical mechanism through I-V characteristic curve fitting and systematic electrical testing. Under low bias voltage, Ag + ions migrated directionally along the electric field gradient toward the Pt electrode, forming a network of discretely distributed Ag nanoclusters inside the MXene layer via reduction reaction. Further analysis shows that these metal clusters can act as quantum potential wells for electron transport, and the conductive channel is established by the thermally assisted quantum tunneling effect under external field excitation, prompting the device transition from HRS to LRS. Notably, when the forward bias voltage is reduced to a nonzero threshold, the device exhibits significant threshold switching characteristics, as the carrier kinetic energy becomes insufficient to overcome the quantum potential barrier, resulting in reversible breakage of the conducting channel. This dynamically balanced quantum transport behavior provides an important physical mechanism underlying the construction of bionic synaptic devices [27].

### Charge Trapping

**Fig. 7:**
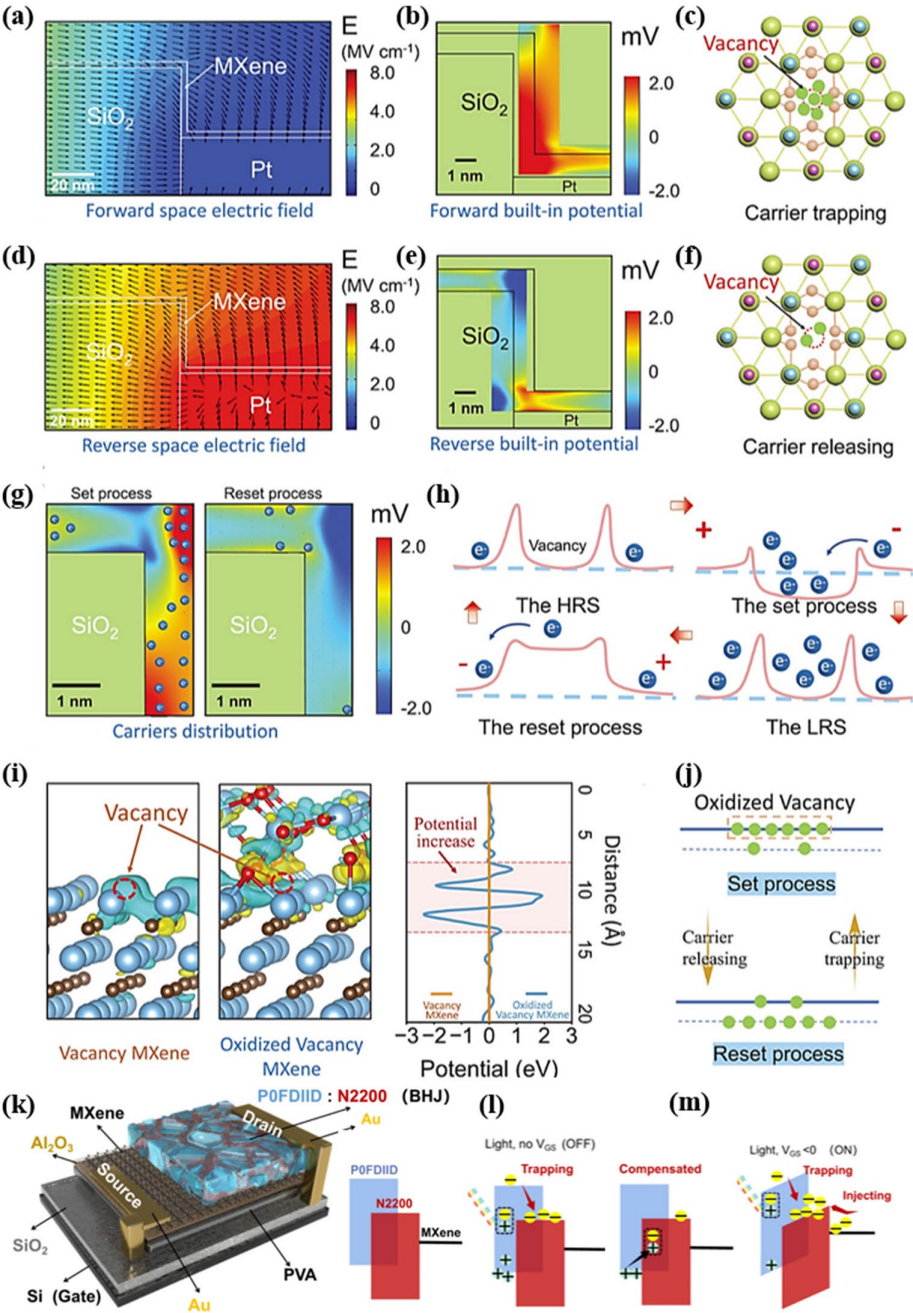
Resistive switching mechanism of the VMMM. **a** Spatial electric field distribution of the VMMM at forward voltage. **b** Built-in potential distribution of monolayer MXene under forward voltage. **c** Carrier trapping state of the oxidized vacancy. **d** Spatial electric field distribution of the VMMM at reverse voltage. **e** Built-in potential distribution of the monolayer MXene under reverse voltage. **f** Carrier releasing state of the oxidized vacancy. **g** Directional modulation effect of the built-in potential on carriers with forward/reverse voltages. **h** Carrier transfer mechanism is modulated by the action of the built-in potential. **i** First-principles calculations of the potential barrier for non-oxidized and oxidized vacancy MXene. **j** Conductive states of the VMMM with the valence and conduction bands under the corresponding external electric fields and built-in potentials. Reproduced with permission [24]. Copyright 2024, Wiley–VCH GmbH [25]. k Structure diagram of VOFET-DR. **l** Schematic diagram of the dynamic changes of charge carriers after the device is input with light pulses when it is not added to V_GS_. **m** Schematic diagram of the dynamic changes of charge carriers after the device is input with light pulses when added to V_GS_

For the neuromorphic device based on the charge capture and release mechanism, a material with rich charge traps is usually used as the functional layer, and when the charge is captured by the charge defects inside the functional layer, the energy level distribution of the material in the functional layer will be changed, which will cause a change in the resistive state of the memristor. Tan et al. reported an oxidized MXene-Ti_3_C_2_T_x_ with monolayer vacancy induction and constructed a memristor with Al/vertical step channel (VSC)-Ti_3_C_2_T_x_/Pt/SiO_2_/Si structure [25]. Based on the vacancy structure model, this study systematically investigates the physical mechanism of this memristor in terms of the spatial regulation mode of the electric field, the microstructure of the vacancy-oxidized MXene-Ti_3_C_2_T_x_, and the asymmetric control of the carrier behavior. During the SET shown in Fig. 7a, the electric field exhibits an abrupt component distortion at the corner of MXene-Ti_3_C_2_T_x_. The simulation analysis in Fig. 7b reveals the existence of a built-in piezoelectric potential as a “built-in gate,” which ensures the reliable maintenance of the carrier capture state facilitated by the piezoelectric properties of the MXene-Ti_3_C_2_T_x_ under the influence of an external electric field [66, 67]. The peaks of the built-in potential are concentrated at the corners of the channel as a significant electric field distortion. As a result, the oxidized vacancy structure can produce a strong binding effect that directs the increase in conductivity. Figure 7c depicts the carrier trapping state of the oxygen vacancy in the presence of an external electric field and a built-in gate, transforming the monolayer of oxidized MXene into a highly conductive channel. In Fig. 7d, the RESET process is shown, where the VSC generates an asymmetric behavior under the influence of the reversed external electric field, causing the carriers to be pulled back. Figure 7e illustrates the reversed electric field in produces the opposite built-in potential and releases the trapped carriers. Figure 7f shows the separation of the carriers from the oxygen vacancies, at which point the device transitions to HRS.

Figure 7g shows the internal modulation effect of the built-in potential on the carriers while directing the regionally oriented motion of the localized carriers during RESET and SET. It forms interactions with the local oxygen vacancy defects, enabling carrier trapping and release. Figure 7h shows the carrier control mechanism involving the built-in potential together with the oxygen vacancy barrier. In the initial HRS state, carriers are hindered by oxygen vacancies, where TiO_2_ forms a barrier that restricts carrier movement and prevents low-carrier transport. During SET, the presence of a localized built-in positive potential attracts carriers to the vacancies, eliminating the electrical block of the monolayer of oxidized MXene-Ti_3_C_2_T_x_ when the vacancies are filled. Thus, the device transitions to LRS. During RESET, the vacancy potential loses its carrier confinement effect with the opposite built-in potential. First-principles calculations further confirm the vacancy potential model, as shown in Fig. 7i. The electronic structure shows that the oxygen vacancies have higher potential barriers than pure MXene-Ti_3_C_2_T_x_ due to the difference in atomic bonding energies. Figure 7j shows that the oxygen vacancies in monolayer MXene-Ti_3_C_2_T_x_ can introduce trapping/de-trapping states between the conduction and valence bands. During the SET process, the built-in potential effect traps the oxygen vacancies and attracts carriers into the conduction band, thus increasing the conductivity. When a RESET voltage is applied, carriers are rapidly withdrawn from the oxygen vacancies and returned to the valence band [68].

In addition, there is a significant modulation of the carrier trapping/de-trapping behavior by light pulses. As shown in Fig. 7k, Gao et al. report an ultra-short channel vertical organic neuromorphic transistor based on MXene network source [69]. In this case, Si/SiO_2_ serves as the substrate, PVA/Al_2_O_3_ is utilized as the charge trapping layer in the dark state, MXene/Au constitutes the network source electrode, the p-type polymer POFDIID and the n-type semiconductor N2200, which form a bulk heterojunction and are used as the charge trapping layer in the light state, and Au serves as the drain electrode. When the gate voltage (V_GS_) is not applied (Fig. 7l), the light pulse triggers the generation of photogenerated excitons in POFDIID and their separation into electron–hole pairs at the POFDIID/N2200 interface. Due to the low LUMO energy level of N2200, electrons are trapped by its traps, while holes contribute to the formation of photocurrents in POFDIID. After the optical pulse ends, the trapped electrons gradually recombine with the holes, resulting in current decay and the manifestation of a short-term memory effect. Subsequently, as shown in Fig. 7m, the application of V_GS_ facilitates efficient separation of photogenerated excitons at the POFDIID/N2200 interface by lowering the Schottky barrier height at the interface between MXene and the POFDIID/N2200 heterojunction. This reduction in non-radiative recombination and increase in feedback strength are observed.

**Fig. 8:**
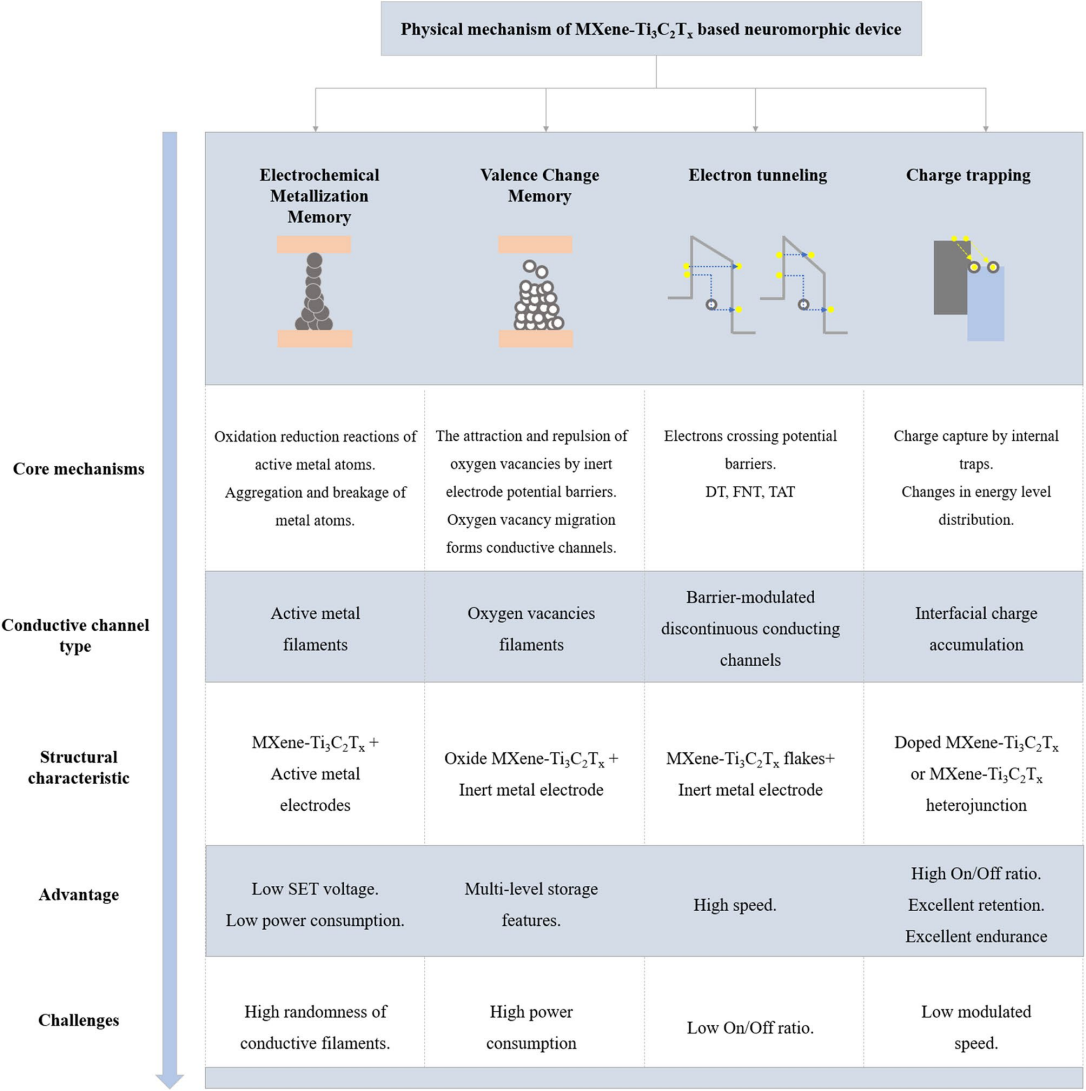
Classification of physical mechanisms of MXene-based neuromorphic devices

Finally, this study systematically comprehends the four physical mechanisms involved in MXene-based memristors, and it develops a multidimensional analysis of these key mechanisms through the comparative framework outlined in Fig. 8. First, the core principles of each mechanism are explained at the microscopic scale, followed by the classification of the construction types of the conducting channels. Subsequently, the typical structural features that correspond to the physical mechanisms are analyzed. Finally, the comparative advantages of each mechanism in terms of response speed, cycling stability, and other performance parameters are summarized, and the key challenges currently faced are highlighted to provide theoretical support and practical guidance for the performance enhancement of MXene-based memristors.

## Performance Enhancement

As an advanced nonvolatile memory device, every leap in the performance of MXene-Ti_3_C_2_T_x_ memristor is directly related to the improvement of data storage capacity, operation speed, and data retention stability. High-performance MXene-Ti_3_C_2_T_x_ memristors not only realize high-speed data transmission but also ensure instant readout and long-term stable data storage [64, 70]. Therefore, researchers have never stopped exploring the performance optimization of MXene-Ti_3_C_2_T_x_ memristors, and constantly seeking new ways to improve performance. These cutting-edge approaches include precise modulation of doping engineering, interface engineering, and structural engineering, aiming to tap the potential of MXene-Ti_3_C_2_T_x_ memristors and lead the future innovation of data storage and computing technologies.

### Interface Engineering

**Fig. 9:**
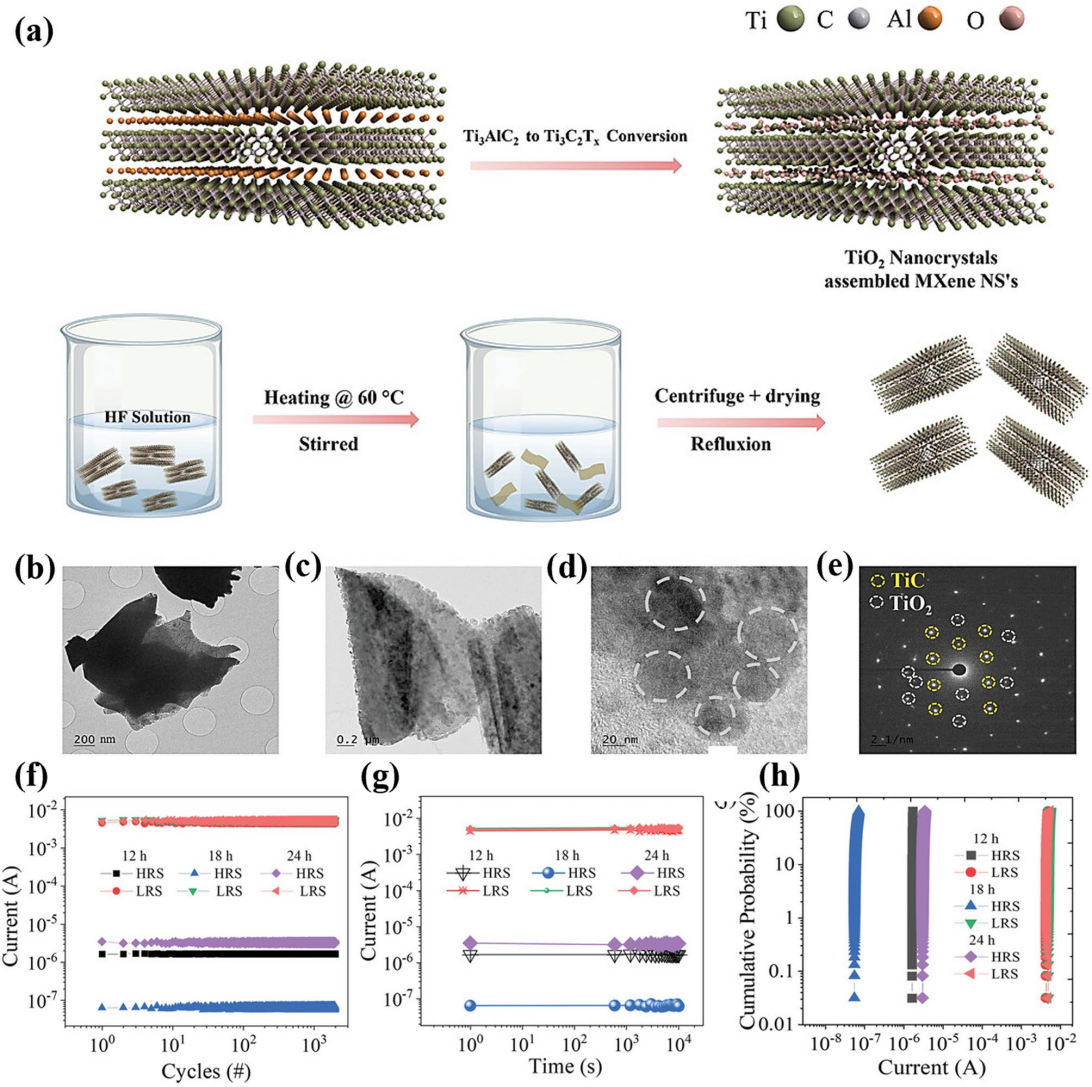
**a** Schematic diagram of the conversion of Ti_3_AlC_2_ to Ti_3_C_2_T_x_ via HF etching and reflux. **b-e** Magnified TEM images of the MXene NSs synthesized through etching at 60 °C for 18 h. **f** Endurance test results, **g** retention test results, **h** cumulative probability distributions for all oxidized MXene NS-based devices. Reproduced with permission [37]. Copyright 2023, Wiley–VCH GmbH [38]

MXene-Ti_3_C_2_T_x_ has the properties of rich surface functional groups and regular arrangement of atomic structure, and its surface can be modified by various chemical methods to change its physical structure and modulate its electronic properties [71, 72]. For example, Mullani et al. describe a technique for controlled etching using hydrofluoric acid that aims to achieve partial oxidation of the surface of MXene-Ti_3_C_2_T_x_ nanosheets [38]. Figure 9a shows a schematic of the method and the synthesis route, where the layered MXene-Ti_3_C_2_T_x_ was first obtained by HF etching of Ti_3_AlC_2_ at 60 °C and refluxed at 40 °C to promote the oxidation sites. Meanwhile, the MXene-Ti_3_C_2_T_x_ nanosheets after 10 h of oxidation were analyzed by transmission electron microscope (TEM) images, as shown in Fig. 9b. In order to analyze the internal oxidation, high-resolution TEM (HRTEM) images of TiO_2_ nanocrystals inside the MXene-Ti_3_C_2_T_x_ nanosheets are presented in Fig. 9c, d. The corresponding crystal structure information is shown in the SAED of Fig. 9e, where the hexagonal arrangement structure of TiO_2_ can be observed along the (0001) axis, which indicates the successful synthesis of titanium oxide nanocrystals. The nonvolatile storage performance of the surface-modified MXene-Ti_3_C_2_T_x_ devices was further evaluated using optimized pulse parameters, as shown in Fig. 9f-h. The results show that the Ag/Ti_3_C_2_T_x_:TiO_2_/Pt memristor exhibits 10^4^ cycles endurance and 10^4^ s resistance retention characteristics, with devices synthesized for different times displaying uniform current accumulation probability distributions. These results indicate that the partially oxidized MXene-Ti_3_C_2_T_x_ memristors have excellent resistance switching performance, good memory retention, and a large memory window for high-density memory and synaptic learning applications [73].

**Fig. 10:**
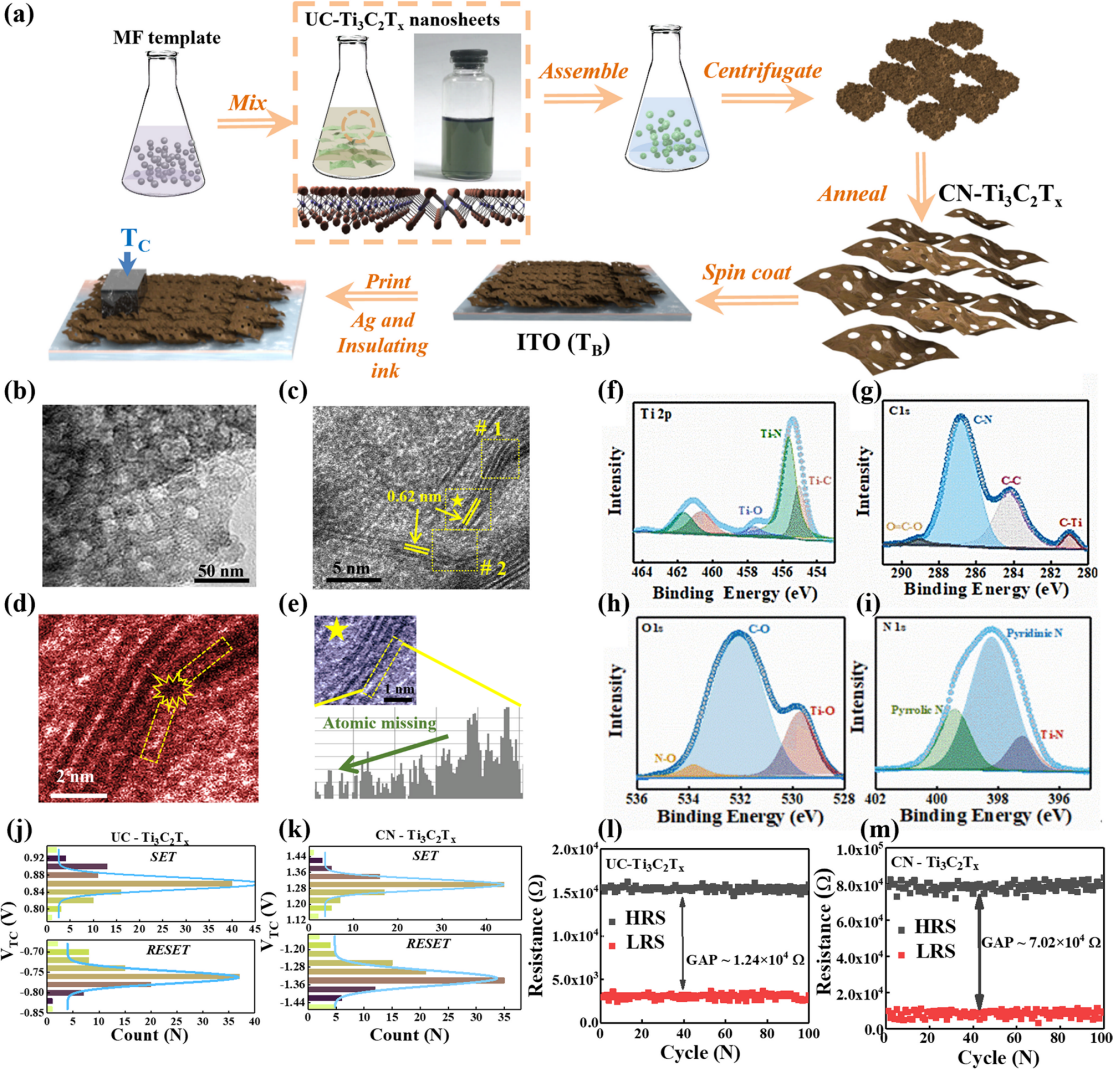
**a** Schematic diagram of the preparation process of CN-Ti_3_C_2_T_x_ nanosheets and NR-S devices. **b** TEM image of CN-Ti_3_C_2_T_x_ nanosheets, with a scale bar of 50 nm. **c** HRTEM image of CN-Ti_3_C_2_T_x_ nanosheets. **d** One of the crumpled positions (#1 and #2). **e** Line profiles analysis of CN-Ti_3_C_2_T_x_. The XPS spectra image of CN-Ti_3_C_2_T_x_ nanosheets: **f** Ti 2*p*, **g** C 1*s*, **h** O 1*s,* and **i** N 1*s*. **j**, **k** Gaussian fitted RESET and SET voltages of two kinds of NR-S devices. **l**, **m** Statistical distributions of high-resistance state (HRS) and low-resistance state (LRS) of two kinds of NR-S devices. Reproduced with permission [29]. Copyright 2021, Wiley–VCH GmbH [30]

Further, Yunfang Jia et al. prepared N-doped crumpled MXene-Ti_3_C_2_T_x_ nanosheet using the melamine–formaldehyde (MF) template method and reported a neuro-receptor-mediated artificial synaptic device [30]. The preparation method is illustrated in Fig. 10a. In this process, the MF template and few-layer MXene-Ti_3_C_2_T_x_ nanosheets were mixed in deionized water. After centrifugation, freeze-drying and vacuum annealing, the N-doped crumpled MXene-Ti_3_C_2_T_x_ nanosheets were obtained. Subsequently, these nanosheets were used as functional layers to fabricate artificial synaptic devices with Ag/CN-Ti_3_C_2_T_x_/ITO structures.

The surface morphology of CN-Ti_3_C_2_T_x_ nanosheets was subsequently observed by TEM, as shown in Fig. 10b-e. Figure 10b shows that the prepared CN-Ti_3_C_2_T_x_ nanosheets have a rougher surface structure. The HRTEM image in Fig. 10c indicates the presence of clear lattice stripes in the CN-Ti_3_C_2_T_x_ nanosheets, with a lattice spacing of 0.62 nm, which is in good agreement with the reported results [74]. In addition, the crumpled regions of CN-Ti_3_C_2_T_x_ nanosheets are also labeled with #1 and #2 in Fig. 10c, and obvious lattice bending can be observed in the enlarged image in Fig. 10d. The line profile analysis in Fig. 10e shows the presence of atomic vacancies in the CN-Ti_3_C_2_T_x_ nanosheets, indicating that the nanosheets were successfully crumpled. The XPS results, shown in Fig. 10f-i, exhibit N 1*s* peaks, which indicate that elemental N was successfully doped into the Ti_3_C_2_T_x_ nanosheets [75]. The RESET and SET voltage distributions of the two NR-S devices were subsequently tested, as shown in Fig. 10j, k.

Strong covalent bonding connections are a commonly adopted approach in the chemical bonding process. Organic and inorganic molecules are tightly anchored to the surface of MXene-Ti_3_C_2_T_x_, thereby constructing a highly stable chemical bonding structure [76, 77]. For example, Yang et al. proposed an innovative surface engineering technique that utilizes the strong ionic interactions between the conjugated backbone and azurite blue (CB) organic ionic materials to modify the surface of MXene-Ti_3_C_2_T_x_ 2D nanosheets, and successfully realized a highly robust MXene-Ti_3_C_2_T_x_ memristor [78]. Specifically, this device with Ag/Ti_3_C_2_T_x_-CB/ITO structure exhibits stable bipolar resistive switching characteristics with a low operating voltage of 0.9 V and a retention time of more than 10^4^ s. In addition, to address the problem of performance degradation after long-term operation, the research team cleverly designed a GO/Ti_3_C_2_T_x_-CB/graphene oxide (GO) two-dimensional heterostructure, which is based on the laminar properties of two-dimensional GO and greatly improves the stability of the performance. Through charge transport modeling and scanning electron microscopy (SEM) analysis, it was found that the 2D GO layer played a key buffering role and effectively suppressed the random diffusion and overgrowth of the conductive filaments. This study not only reveals the broad prospect of organic ion-electronic modification strategy for the preparation of high-performance MXene-Ti_3_C_2_T_x_ memristors, but also provides valuable guiding ideas to promote the wide application of novel 2D nanomaterials in the field of nonvolatile memory.

### Doping Engineering

**Fig. 11:**
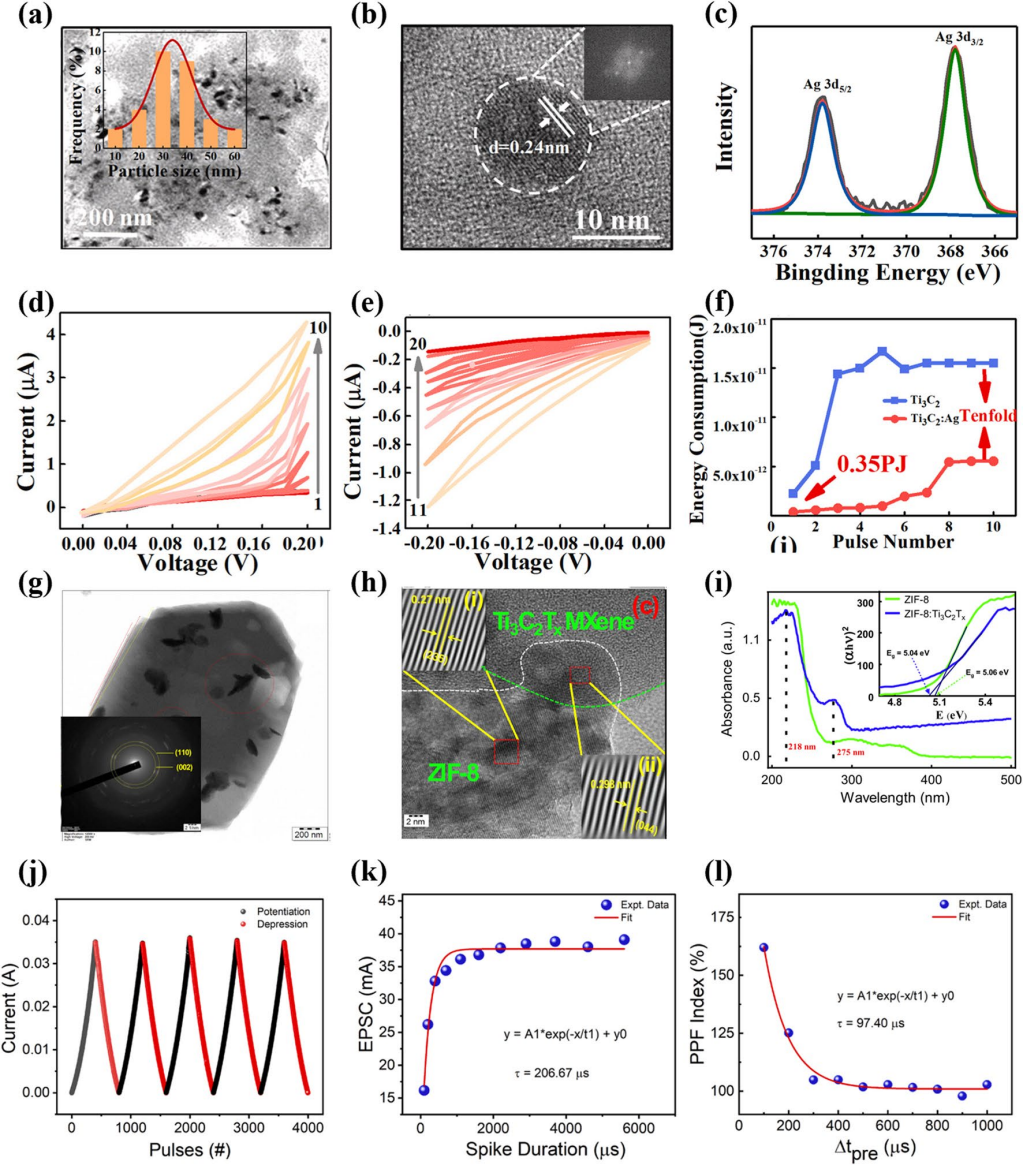
**a** Typical TEM image of the Ti_3_C_2_T_x_:Ag sample, with Gauss fitting of particle size distribution shown in the inset. **b** High-resolution TEM image depicting Ag nanoparticles within Ti_3_C_2_T_x_. The inset displays the FFT pattern of Ag particles. **c** XPS analysis results for Ag. **d, e** I-V curves of the Al/Ti_3_C_2_T_x_:Ag/Pt device under positive and negative voltage stimuli, respectively. **f** Energy consumption variation comparison between Ti_3_C_2_T_x_ and Ti_3_C_2_T_x_:Ag devices. Reproduced with permission [32]. Copyright 2020, Elsevier Ltd. **g**, **h** HRTEM morphologies of ZIF-8Ti_3_C_2_T_x_, with the insert showing the SAED pattern of ZIF-8: Ti_3_C_2_T_x_. **i** UV–visible absorption spectrum (inset: Tauc plot for Eg calculation) of ZIF-8 and ZIF-8:Ti_3_C_2_T_x_ nanocomposite. **j** Potentiation–depression characteristics. **k** EPSC properties. **l** PPF properties, both mimicked by Ag/ZIF-8:Ti_3_C_2_T_x_/FTO RS device. Reproduced with permission [77]. Copyright 2024, Elsevier B.V [80] :

Usually, the formation of the conductive channel in the memristor is often accompanied by a large degree of randomness, which leads to the device being prone to instability during the setting process, thus affecting its durability and repeatability [79]. Therefore, it is particularly important to effectively standardize the formation path of the conductive channel to ensure that the memristor can exhibit stable and controllable electrical characteristics. The majority of researchers are committed to exploring innovative methods and strategies to achieve precise regulation of the conductive channel in the memristor, which will enable neuromorphic devices to show more excellent performance and stability in practical applications. Yan et al. proposed a facile method for doping Ag nanoparticles to improve the electronic performance of a 2D MXene-Ti_3_C_2_T_x_-based memristor [32]. Figure 11a shows a TEM image of the MXene-Ti_3_C_2_T_x_:Ag nanosheets, the inset image displaying a more consistent diameter distribution and size of the silver nanoparticles, which follows a Gaussian distribution. The magnified imaging of the silver nanoparticles is shown in Fig. 11b, where it can be observed that the silver nanoparticles have well-defined boundaries and circular characteristics.

The inset of Fig. 11b displays the fast Fourier transform (FFT) image of the silver nanoparticles, revealing a lattice spacing is 0.24 nm, which is consistent with the single-crystal property of the silver nanoparticles This is further corroborated by the XPS energy spectrum shown in Fig. 11c [81]. Compared to the Al/Ti_3_C_2_T_x_/Pt devices, the Al/Ti_3_C_2_T_x_:Ag/Pt devices exhibit controllable bi-directional continuous resistive switching behavior, as illustrated in Fig. 11d, e, and the energy consumption is only 0.35 pJ under the single-pulse spike (Fig. 11f). This work provides a facile method to improve the neuromorphic properties of MXene-Ti_3_C_2_T_x_ memristors, which will significantly contribute to the diversification of 2D materials in the field of neuromorphic chips and greatly enhance the utility of MXene-Ti_3_C_2_T_x_ materials.

Organic–inorganic hybrid materials exhibit great potential for applications in the field of electronic devices. Organic materials possess flexible structures and rich functionalities, while inorganic materials offer stable backbones and excellent electrical and thermal conductivity. The combination of organic and inorganic materials not only broadens the research boundaries of materials science, but also greatly enriches the application prospects of electronic devices. In particular, the fusion of organic and inorganic materials also contributes to the crucial enhancement of the electrical properties of MXene-Ti_3_C_2_T_x_ artificial synapses. Abdullah et al. presented a nanocomposite based on ZIF-8 and MXene-Ti_3_C_2_T_x_ for the preparation of artificial synaptic devices [80]. Figure 11g shows MXene-Ti_3_C_2_T_x_ nanosheets doped with ZIF-8 particles, with the inset showing the electron diffraction pattern of ZIF-8:Ti_3_C_2_T_x_ [82]. Figure 11h shows two regions with different lattice spacing in the ZIF-8:Ti_3_C_2_T_x_ nanosheets. Figure 11i exhibits the absorption spectra of ZIF-8 and ZIF-8:Ti_3_C_2_T_x_ within the range of 200–500 nm. The band gap value of ZIF-8 material was determined to be 5.06 eV using the Tauc plot. The presynaptic, postsynaptic, and synaptic gaps were simulated using the top electrode (Ag), bottom electrode (FTO), and ZIF-8:Ti_3_C_2_T_x_ switching layer, respectively. Figure 11j shows the potential enhancement and inhibition characteristics of the Ag/ZIF-8:Ti_3_C_2_T_x_/FTO device under continuous voltage bias from 0 to 2.5 V. These characteristics are associated with positive and negative changes in synaptic weights, effectively simulating potential enhancement and inhibition characteristics similar to those observed in the human brain. As shown in Fig. 11k, the Ag/ZIF-8:Ti_3_C_2_T_x_/FTO device efficiently simulates excitatory postsynaptic current (EPSC). The EPSC current exhibits a nearly steep increase when the duration of the presynaptic spike is less than 2000 μs and reaches saturation thereafter. In addition, paired-pulse facilitation (PPF) experiments were also performed, as depicted in Fig. 11l. The PPF index decreased gradually with the increase in pulse interval, indicating that the Ag/ZIF-8:Ti_3_C_2_T_x_/FTO memory device could mimic the short-term memory properties of the human brain and could be utilized for the development of neuromorphic devices.

### Structural Engineering

**Fig. 12:**
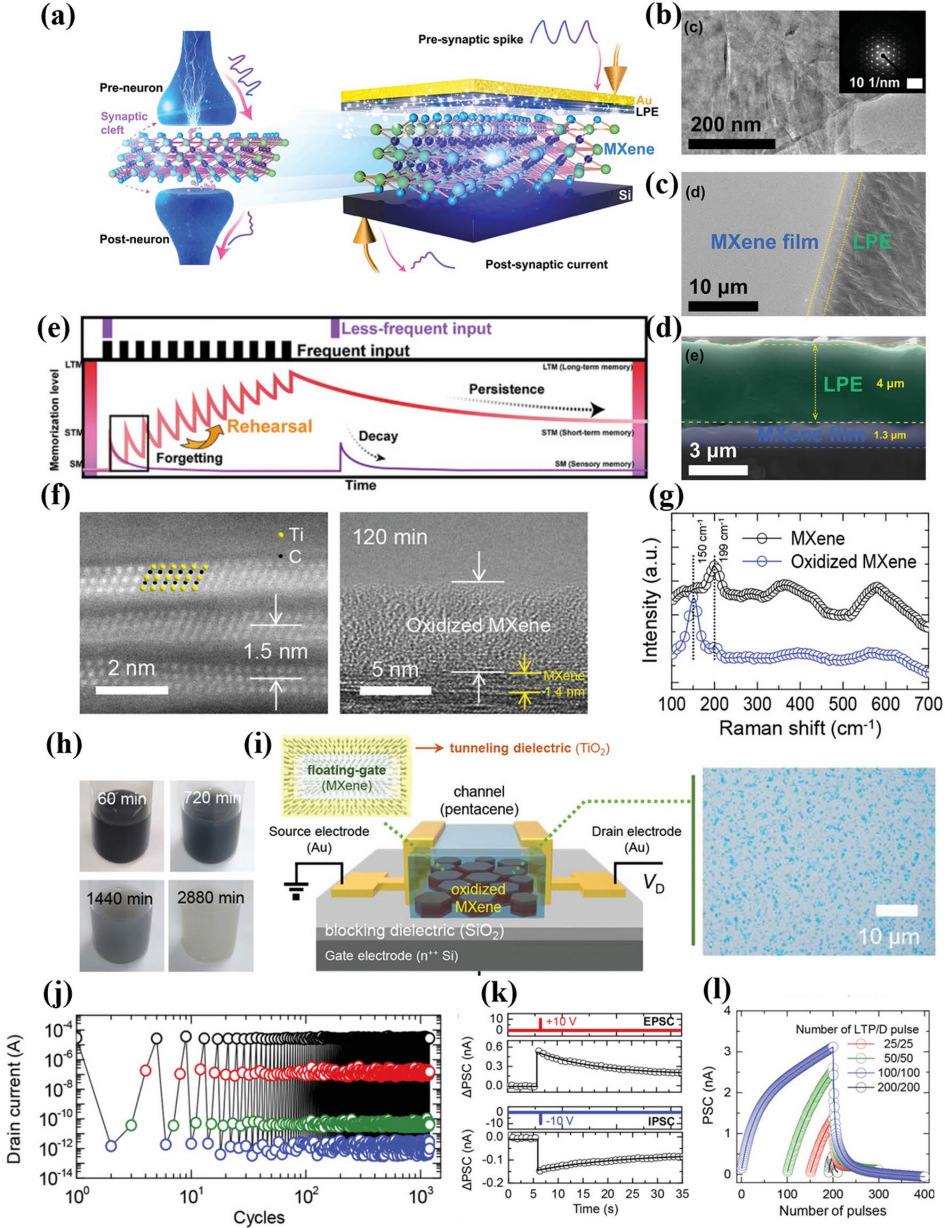
**a** Schematic illustration of biological synapse and MXene-Ti_3_C_2_T_x_ artificial synapses. **b** TEM image of MXene-Ti_3_C_2_T_x_, with the inset showing the SAED pattern. **c** SEM image of MXene/LPE film. **d** Cross-section image of MXene/LPE film. **e** Simplified memorization model inspired by the multistore model. Reproduced with permission [17]. Copyright 2020, Wiley-VCH GmbH. **f** Cross-sectional STEM images of pristine MXene-Ti_3_C_2_T_x_ and oxidized MXene-Ti_3_C_2_T_x_. **g** Raman spectra of pristine MXene-Ti_3_C_2_T_x_ and oxidized MXene-Ti_3_C_2_T_x_. **h** Photographic images of MXene-Ti_3_C_2_T_x_ solution at different stages of oxidation. **i** Schematic illustration of NFGTMs. The right panel displays the optical microscopy image of the oxidized MXenes on the SiO_2_ blocking dielectric layer. **j** Cyclic stability of NFGTMs over 1000 programming/erasing cycles. **k** EPSC and IPSC responses of NFGTMs to VWC spike with a magnitude of ±10 V and duration (td) of 10 ms. **l** LTP/LTD characteristic curves as a function of number of pulses. Reproduced with permission [36]. Copyright 2020, WILEY–VCH Verlag GmbH & Co. KGaA, Weinheim [37]

The skillful utilization of the dielectric layer to regulate ion transport channels is of great significance in the preparation of highly stable neuromorphic devices. As a barrier layer, the dielectric layer significantly minimizes the direct exposure of MXene-Ti_3_C_2_T_x_ to the external environment, effectively mitigating environmental interference and thus substantially enhancing the long-term stability and reliability of the memristor, for example, the optimization of key performance indicators including significantly reducing the operating voltage and increasing the number of resistive states. Through the dielectric layer coupling technology, the memristor not only demonstrates stable resistive switching characteristics but also concurrently exhibits substantial capacitive effects. This combination of dual characteristics significantly broadens the application scope of neuromorphic devices [83, 84]. Wei et al. reported an artificial synaptic device with MXene-Ti_3_C_2_T_x_ nanosheets coupled to a solid lithium polymer electrolyte (LPE) layer, as shown in the Au/LPE/MXene-Ti_3_C_2_T_x_/Si structure in Fig. 12a [17]. The TEM image of the MXene nanosheets, shown in Fig. 12b, indicates typical hexagonal symmetry properties in its SAED pattern. The SEM image in Fig. 12c displays the successful coupling of this polymer electrolyte to the MXene-Ti_3_C_2_T_x_ nanosheets, while the cross-sectional image in Fig. 12d reveals the thicknesses of both the LPE and MXene-Ti_3_C_2_T_x_ nanosheets. When multiple stimuli are applied to Au/LPE/MXene-Ti_3_C_2_T_x_/Si, as shown in Fig. 12e, the memory level of the device transitions from short-term memory (STM) to long-term memory (LTM) [85], but STM decays easily and thus needs to be transformed to LTM through multiple voltage stimulations. With the help of this special dielectric layer coupling technique, the device boasts an ultra-low power consumption of 460 fW and an ultra-small response voltage of ± 80 mV, which even surpasses that of biological synapses. In addition to dielectric layer coupling, full-wrap coupling is also a crucial method for enhancing the stability of MXene-Ti_3_C_2_T_x_-based memristors. Lyu et al. prepared MXene-TiO_2_ core–shell nanosheets by precisely controlling the surface oxidation of MXene. The MXene-TiO_2_ core–shell nanosheets were subsequently sandwiched between an organic semiconductor layer and a SiO_2_ barrier dielectric layer as a floating gate and tunneling layer in nano-floating-gate transistor memory (NFGTM) by a low-cost and environmentally friendly water-based oxidation process [37]. Figure 12f shows the cross-sectional scanning transmission electron microscopy (STEM) images of pure MXene-Ti_3_C_2_T_x_ nanosheets and MXene-TiO_2_ core–shell nanosheets, depicting the atomic structure of the MXene-Ti_3_C_2_T_x_ layer and the presence of the oxide layer. The Raman spectra of both samples are shown in Fig. 12g and reveal a new peak at 150 cm−1 corresponding to TiO_2_, indicating the conversion of Ti_3_C_2_T_x_ to TiO_2_ during the oxidation reaction. Figure 12h displays photographic images of the MXene solution at different stages of the oxidation process, with the color change from black to white confirming the gradual oxidation of MXene as the reaction time increases. Figure 12i presents schematic of MXene-NFGTM device and illustrates the storage performance of the device when an optimal oxide thickness of 6.2 nm is achieved. Figure 12j shows 1000 programming/erasing processes of the device with no significant performance degradation, indicating excellent operational endurance. The synaptic functions of the MXene NFGTMs are shown in Fig. 12k, l.

Figure 12k shows the results of the EPSC and inhibitory postsynaptic currents (IPSC) response of the NFGTM under pulse spikes with amplitude ± 10 V and pulse width of 10 ms, where the postsynaptic voltage (V_post_) is fixed at + 5 V. The PSC shows a current jump amplitude of 0.56 nA when a positive pulse of + 10 V is applied, and decreases to 0.16 nA after a negative pulse of −10 V is applied. In this case, the PSC did not return to the initial level after 30 s, indicating that a pulse with an amplitude of ± 10 V and a duration of 10 ms could promote long-term plasticity. The long-term potentiation (LTP) and long-term suppression (LTD) characteristics of NFGTMs were further analyzed by varying the number of pulses under the condition of pulse spiking with an amplitude of ± 10 V, as shown in Fig. 11l. After increasing the number of pulses from 100 to 200, the G_max_/G_min_ further increased, and the power consumption per single-pulse spike was only 3.8 pJ. This power consumption, which is close to that of biological synapses in the human brain, suggests that full-wrap coupling can make an important contribution to enhancing the performance of artificial synaptic devices.

**Fig. 13:**
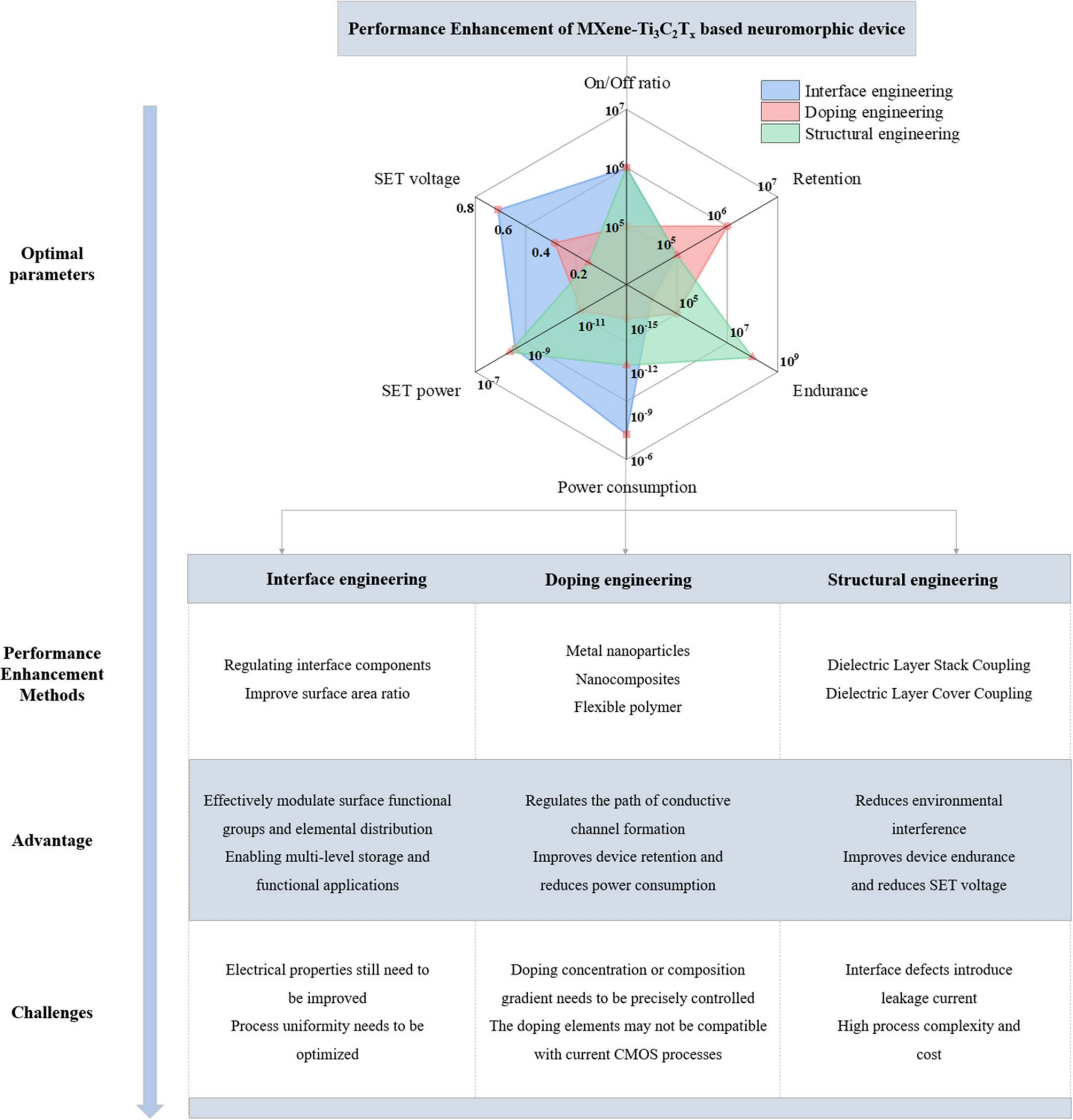
Classification of performance enhancement methods of MXene-based neuromorphic devices

Finally, as shown in Fig. 13, we systematically summarize the performance enhancement strategies for MXene-Ti_3_C_2_T_x_-based neuromorphic devices, covering three types of core methods, namely interface engineering, doping engineering, and structure engineering. These methods significantly enhance the multilevel storage capability, retention properties, and endurance of the devices by precisely tuning the surface functional groups, elemental distributions, and conductive path formation mechanisms, as well as reducing the SET voltage and power consumption, and enhancing the resistance to environmental disturbances. For instance, from the optimal parameters in the radar chart, it can be seen that doping engineering can significantly reduce the energy loss of the device. Structural engineering is used to effectively improve device durability and reduce SET voltage. However, some challenges such as doping concentration control, lack of process uniformity, interfacial leakage current, and compatibility with conventional CMOS processes still need to be overcome, and the high complexity of the preparation process increases the cost of the application. Therefore, future research needs to further synergistically optimize the material intrinsic properties and interface design to promote the industrialization of high-performance neuromorphic devices.

## Cutting-Edge Computing

**Fig. 14:**
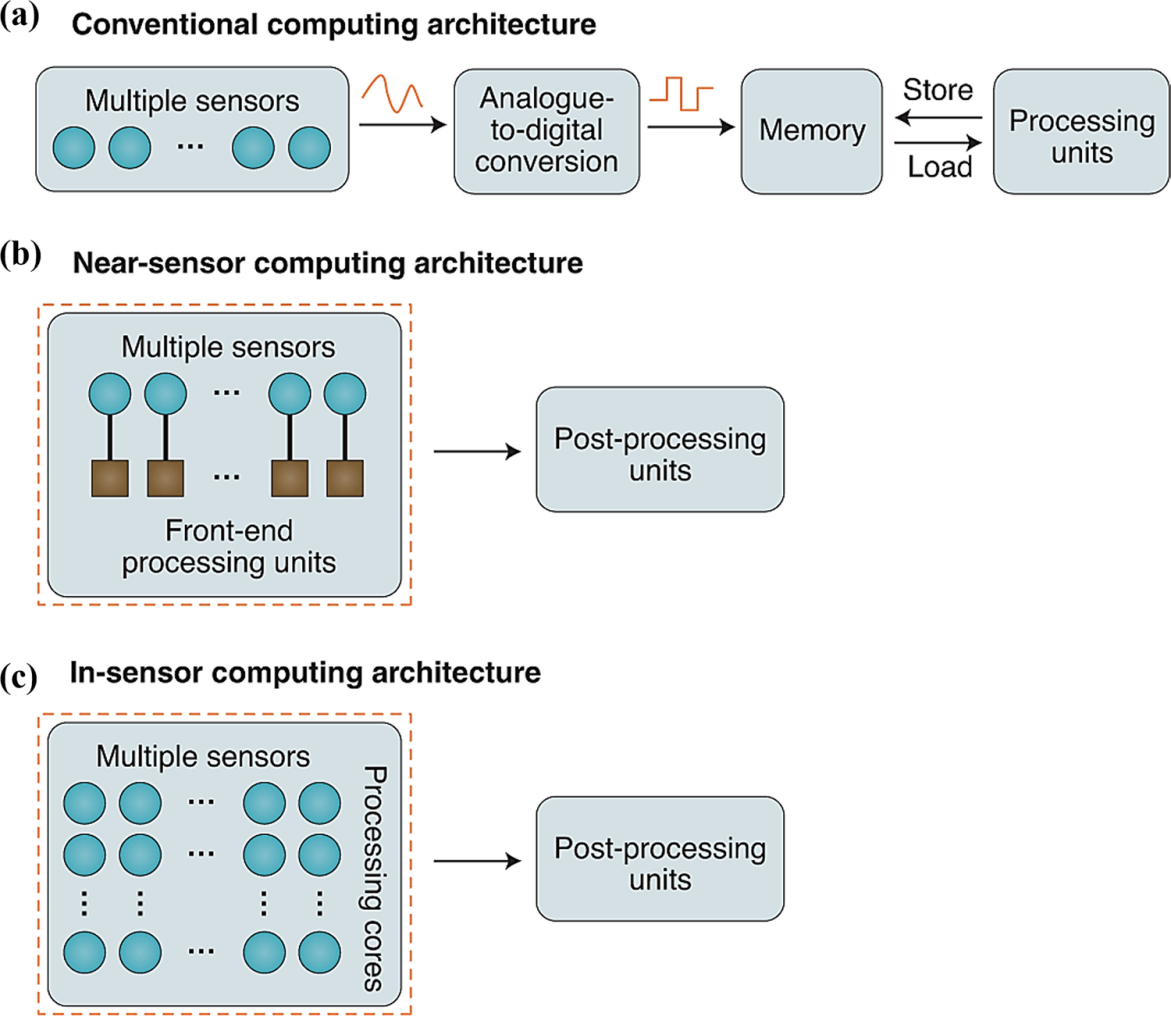
**a** Conventional sensory computing architecture. **b** Near-sensor computing architecture. **c** In-sensor computing architecture. Reproduced with permission [86]. Copyright 2020, Springer Nature Limited

Within traditional computer architectures (Fig. 14a), analog sensing signals are converted to digital signals via analog-to-digital converters (ADCs), and the data are stored temporarily in a memory unit, from which it is transferred to the CPU for processing. This approach, which relies on data conversion and transmission paths, inevitably introduces high levels of energy consumption and significant response delays. In contrast, the near-sensor computing architecture shows significant advantages. In this architecture (Fig. 14b), processing units are deployed close to the sensors to perform specific tasks directly on the sensor side. This design not only optimizes the interface between the sensor and the processing unit, but also cuts down the amount of unnecessary data transmission, which fundamentally improves the system efficiency. The near-memory computing architecture optimizes system efficiency by deploying processing units near sensors to execute specific tasks directly at the sensor level. This approach effectively optimizes interface design and reduces redundant data transmission. Its core philosophy lies in shortening the physical distance between computing and memory units rather than pursuing full integration, compressing data pathways to minimize transmission latency and energy consumption. This makes it particularly suitable for scenarios requiring high-frequency data interactions but with relaxed integration requirements. However, the spatial separation of sensors and processors leads to an architecture that is still unable to fully circumvent the physical limitations of the data conversion and transmission chain, which to a certain extent constrains further improvements in energy efficiency. Furthermore, the emergence of in-sensor computing architecture has pioneered a new paradigm of deep collaboration between perception and computation (Fig. 14c). By processing captured raw information locally, through self-adaptive sensors or interconnected sensor networks, it breaks through traditional interface bottlenecks between sensors and processors, achieving the seamless integration of physical sensing and intelligent computing. Leveraging its highly efficient on-device data processing capabilities, this architecture excels particularly in handling large-scale parallel computing tasks and deep learning operations. While eliminating redundant data transmission, it significantly enhances real-time performance, thereby offering superior solutions for high-performance computing, edge computing, and similar application scenarios. Inspired by this trend, MXene-Ti_3_C_2_T_x_-based memristor technology is rapidly emerging in the field of in-memory computing, providing a new physical foundation and technical path for building more efficient, compact and responsive sensing and computing systems [86, 87].

### In-Device Simulation Strategy

**Fig. 15:**
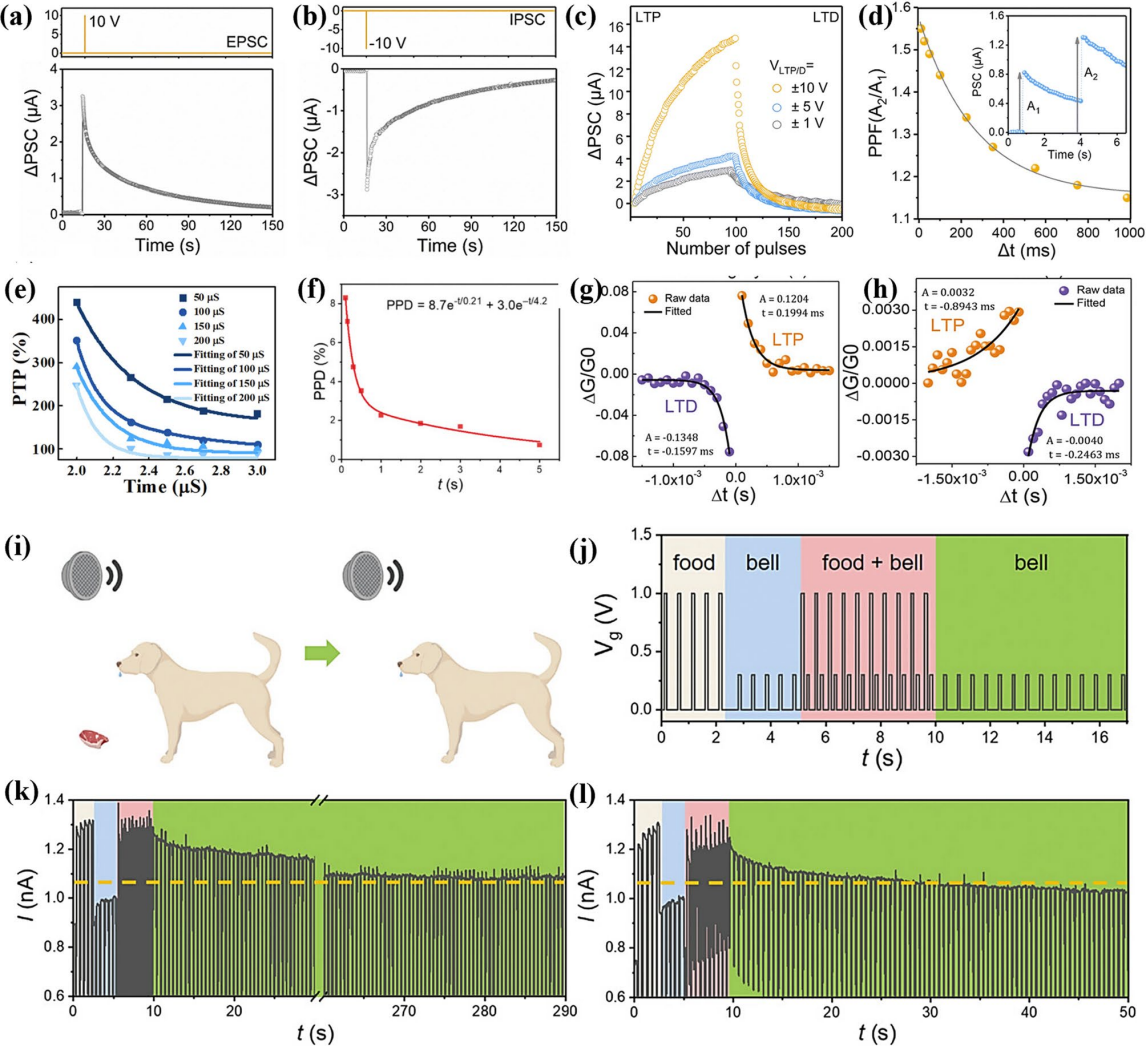
**a** EPSC and **b** IPSC responses of the memory device to V_WC_ spikes with a magnitude of ± 10 V and duration (td) of 10 ms. **c** LTP/LTD behaviors for synaptic operation in neuromorphic computing. **d** PPF index-Δt curve indicating short-term plasticity characteristics of the synaptic device. Reproduced with permission [88]. Copyright 2024, The Royal Society of Chemistry. e PTP fitted with different pulse duration. Reproduced with permission [31]. Copyright 2020 Elsevier Ltd. [32]. f PPD characteristics of a floating-gate synaptic transistor (gate pulse amplitude: − 1 V). Reproduced with permission [91]. Copyright 2024 Wiley–VCH GmbH. **g** Antisymmetric Hebbian (ASH) and **h** antisymmetric anti-Hebbian (ASAH) learning rules mimicked by MemOSC device. **i** Schematic diagram of Pavlov’s dog experiment. **j** Simulating Pavlov’s experiment using a floating-gate synaptic transistor. **k** Pavlovian associative learning achieved by floating-gate synaptic transistor. **l** Pavlovian associative learning behavior of floating-gate synaptic transistor under bending with a curvature radius of 6 mm

To advance the realization of near-sensor computing and in-sensor computing technologies, researchers have deeply explored the core functional aspects of the devices and designed an experimental scheme based on the electrical stimulation of MXene-Ti_3_C_2_T_x_ artificial synaptic devices to mimic the dynamic transport mechanism of Ca^2+^ ions between the synaptic gaps in the neural system, to realize the sensing, learning, and memory functions of the artificial synapses. This process not only involves a deep understanding of the principles of biological nerve conduction, but also integrates advanced electronic engineering techniques, aiming to validate and optimize the potential and feasibility of the devices in simulating synaptic plasticity and realizing functional storage, which provides a strong support for the construction of highly biomimetic neuromorphic computing systems. Wang et al. reported a nonvolatile transistor memory (NVTM) device with hybrid nanocomposites (defined as the MXP) as the floating-gate (FG) layer, which was realized by simply intercalating poly(3-trifluoromethylstyrene) nanoparticles (PTF) into MXene-Ti_3_C_2_T_x_ nanosheets [88]. Notably, the MXene-Ti_3_C_2_T_x_-based floating gate not only achieves retention performance comparable to conventional graphene-based transistors (exceeding 10^5^ s) [89], but also exhibits a wider memory window (greater than 47.8 V), attributed to its higher density of surface functional groups and stronger charge trapping capability [90]. As shown in Fig. 15a, b, after applying a gate voltage with an amplitude of ± 10 V and a pulse width of 10 ms, respectively, both enhanced and inhibited neural response signals can be realized, and the postsynaptic currents (PSCs) return to the initial level only after 100 s, which demonstrates that the devices can realize the simulation of EPSCs and IPSCs. Figure 15c is generated by continuously applying 100 pulse spikes (V_WC_ = ± 1, ± 5, and ± 10 V, td = 10 ms, Δt = 0.5 s) of stimuli during enhancement and inhibition. This characterization demonstrates the feasibility of the device to achieve LTP and LTD, which is crucial for achieving high learning accuracy in neuromorphic computation. In addition, PPF was measured at pulse voltages with different pulse intervals (Δt), as shown in Fig. 15d. The PPF-Δt curves can be fitted by the double-exponential function of Eq. (5):

5 $$\text{PPF}=1+{C}_{1}{\text{e}}^{\left(-\frac{\Delta t}{{\tau }_{1}}\right)}+{C}_{2}{\text{e}}^{\left(-\frac{\Delta t}{{\tau }_{2}}\right)}$$

where C_1_ and C_2_ are the initial facilitation magnitudes, and τ_1_ and τ_2_ represent the rapid and slow relaxation time constants, respectively.

Wang et al. prepared a memristor with Al/Ti_3_C_2_T_x_:Ag/Pt structure and realized another important excitatory synaptic property (post-tetanic potentiation (PTP)) [32] which is distinct from the second EPSC peak selected for PPF and the tenth EPSC peak typically chosen for PTP studies. Figure 15e demonstrates that the Al/Ti_3_C_2_T_x_:Ag/Pt memristor successfully mimics the behavior of PTP. Paired pulse depression (PPD) is a typical form of short-term synaptic plasticity found in biological neurons, which is manifested as synaptic inhibitory properties following two consecutive stimuli. As shown in Fig. 15f, Zhu et al. reported a flexible MXene floating-gate synaptic transistor [91], where it can be observed that the PPD value gradually decreases from 8% to 0.8% with increasing pre-pulse interval. This result underscores the biologically similar synaptic plasticity exhibited by MXene floating-gate synaptic transistors. Additionally, Nirmal et al. developed an organic artificial synapse with flexible, transparent electrodes based on a multilayer hybrid MXene/Ag/MXene structure [92]. They simulated the most crucial STDP rules in Hebbian learning by modulating the pulse interval between presynaptic and postsynaptic spikes, achieving both antisymmetric Hebbian (ASH) and antisymmetric anti-Hebbian learning within the STDP framework, as illustrated in Fig. 15g, h. In computational neuroscience, these types of STDP rules can be fitted with the exponential function in Eq. (6):

6 $$\Delta W=A\times {e}^{\left(\frac{-\Delta t}{\tau }\right)}$$

where A is the scaling factor, τ is the time constant, and ΔW is a change in the synaptic weight.

In addition to conditional in-device simulations, functional in-device simulations are often explored. Zhu et al. reported a flexible MXene-Ti_3_C_2_T_x_ floating-gate synaptic transistor and successfully modeled the important Pavlovian conditioned reflex during associative learning, which is important in both physiology and psychology [91]. Figure 15i illustrates a Pavlovian dog experiment, where through repetitive stimulation, the dog learns to associate the ringing of a bell with food, ultimately leading to salivation triggered solely by the bell sound. The neuromorphic device simulates the presence of food and bell ringing by varying the amplitude of presynaptic pulses. As shown in Fig. 15j, presynaptic pulses with amplitudes of −1 and −0.3 V corresponded to the “food” signal and the “bell” signal, respectively. A postsynaptic current of 1.08 nA was defined as the threshold for eliciting the “salivation” response, indicating activation of the dog’s salivary glands. Figure 15k, l demonstrates the performance of the MXene floating-gate synaptic transistor during the simulation of Pavlovian conditioned reflexes. The experimental results reveal that the device exhibits distinct postsynaptic currents based on the type and combination of presynaptic pulses, underscoring the feasibility of MXene-based artificial synaptic devices in simulating complex cognitive processes, including learning and memory.

### Out-Device Simulation Strategy

With the rapid progress of neuromorphic computing technology, multifunctional near-sensor computational sensory neurons and integrated in-sensor computational devices based on the out-device simulation strategy are flourishing at an unprecedented speed and show great application potential and unique advantages in large-scale neuromorphic sensory circuitry construction as well as advanced human–computer interaction systems. These innovative technologies not only greatly improve the data processing efficiency and intelligent response capability, but also lay a solid foundation for realizing a more natural and efficient human–computer interaction experience.

#### Tactile Perception

**Fig. 16:**
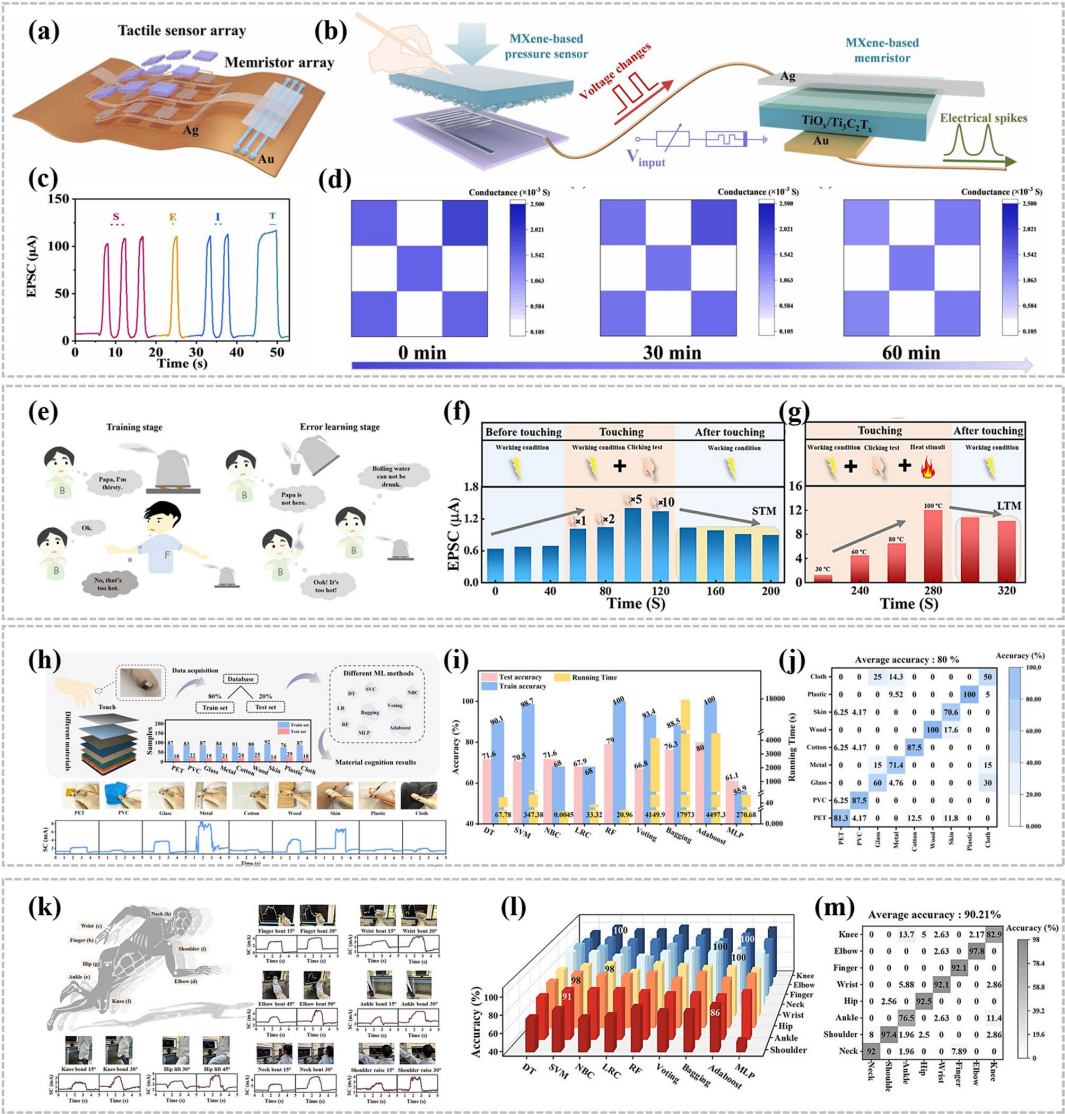
**a** Schematic diagram of all MXene-based near-sensor computing systems consists of a 3 × 3 flexible pressure sensor array and a 9 × 3 memristor array. **b** Schematic diagram and connection circuit diagram of a sensory neuron. **c** Tactile signal is transmitted from sensor unit to artificial neural synapse unit. EPSC signals were generated by artificial sensory neurons when tactile stimulus was applied representing the signals of “SEIT” in Morse code. **d** Handwritten letters are read by the sensor array to convert the signal into a voltage signal input to the artificial synaptic array. Synaptic weight mapping of 3 × 3 artificial synaptic array. Reproduced with permission [31]. Copyright 2024, Elsevier Ltd. **e** Schematic diagram of operant conditional flex behavior. The excitatory postsynaptic current (EPSC) in **f** the first training stage and **g** the second personal experience stage. The guidance from father and the burning pain from hot water are emulated by the clicking test and heat stimuli, respectively. Reproduced with permission [26]. Copyright 2022, Elsevier Ltd. **h** Scheme for material cognition including data acquisition of SCs, the training, testing, and evaluation of models. The prepared ANS device is mounted on the glove, and contacted nine kinds of materials in the same manner are shown in the first line. **i** Evaluations of different ML methods for material cognition, by comparing train accuracy, test accuracy, and running time. **j** Confusion matrix for the accuracy of material cognition classification using the AdaBoost algorithm. **k** Distribution of joints equipped with ANS devices. **l** Accuracies of nine ML approaches for recognizing the motion degrees at eight joints, and the highest ones are marked. **m** Confusion matrix for classifying the position of the moving joint when using the AdaBoost algorithm

Huang et al. report an all-MXene-based flexible sensory neuron fabricated by a room-temperature energy-efficient controlled oxidation preparation technique for the implementation of simulated perceptual behaviors [31]. The structure is shown in Fig. 16a, where a flexible MXene-based pressure sensor array converts an external physical signal into an action potential, and then, the stimulus signal is used as an input to the flexible artificial synapse array for data storage and processing. By adjusting the time, amplitude, and interval of the pressure input on the sensor array, the pulse input parameters of the artificial synapse array were controlled to realize the modulation of the EPSC output of the memristor array, as shown in Fig. 16b. Subsequently, Morse code is used as an example to demonstrate the application of this synaptic array. The textual message is encoded and fed as an electrical signal to the memristor unit, where a fast dynamic pressure is loaded on the sensor to generate the dot signal in Morse code and a static pressure of predetermined duration is applied to the sensor to generate the dash signal. The message is decoded by collecting the EPSC output value of the memristor. Figure 16c shows the EPSC results of the output of the memristor unit after inputting “SEIT” from the sensor unit, which indicates the potential of this sensory memory system for realizing learned behaviors. In addition, after writing the word “X” by pressing the 3 × 3 sensor array, the conductance value of the artificial synapse could be read by applying a small reading voltage of 0.10 V, as shown in Fig. 16d. After a period of time, the conductance mapping of the memristor can be read by the same reading voltage, and the information stored in the memristor array can also be obtained.

Associative learning processes are also an important part of neuroscience research, which involves important operant conditioning behavior [93]. Wang et al. reported an afferent nervous system (ANS) device consisting of the MXene-Ti_3_C_2_T_x_ memristor and ionic conductive elastomer (ICE) [26]. The ANS device can simulate the classical learning model through “trial-and-error learning,” using real clicking actions and thermal stimuli as external inputs to represent the father’s body language and boiling water, as shown in Fig. 16e. As shown in Fig. 16f, under the optimized operating conditions, the initial value of the EPSC is approximately 0.6 µA. After the clicking experiment, the EPSC is significantly enhanced and remains constant after 10 clicks. When the clicking action is withdrawn, the EPSC decreases to approximately 1.0 µA, which can be regarded as a state of STM. The change rate of the EPSC during the first training indicates that the ANS device can recognize the clicking action and gradually develop an STM state. In addition, an experiment simulating drinking boiling water was conducted using thermal stimulation, as shown in Fig. 16g. It was found that when the temperature was increased from 30 to 100 ºC, the EPSC increased from 1 to 12 µA and remained stable at 12 µA, resembling the LTM observed in the “trial-and-error learning” experiment. Overall, the proposed device can establish a connection between artificial synapses and real external stimuli, thereby opening up more possibilities f for the implementation of in-sensor computing with artificial synapses. As shown in Fig. 16h [94], Ren et al. attached the ANS device to a glove and contacted nine different materials, including PET, glass, metal, cotton, wood, skin, polyvinyl chloride (PVC), plastic, and cloth (consisting of cotton and nylon), through their fingers. Synaptic currents were captured during the contact, and a database was created. Various machine learning algorithms were then used to train the material cognition model. Experiments demonstrated that the material cognition model, using the AdaBoost algorithm, achieved a classification accuracy of 80%, as illustrated in Fig. 16i, j. Based on the aforementioned work, the integration of multimodal sensing and intelligent algorithms of the bionic ANS device was extended to the limb joints for additional motor cognition tests. By distributing the prepared devices on each joint, as shown in Fig. 16k, and recording the triggered synaptic currents signals, the ANS device can capture not only the obvious movements occurring on the limb joints, but also the relatively small changes in the degree of movement of the same joint. Nine machine learning methods were employed to explore the motion recognition of eight joints and their classification, as shown in Fig. 16l, m. The classification accuracy reached as high as 86% or even 100% at the neck, finger, elbow, and knee positions. The application of the proposed ANS device extends to nearly all body joints, and it can be considered a promising candidate for realizing in-sensor computing. Notably, the architecture of integrating physical signal conversion, data storage, and pulse modulation inside the MXene neuromorphic device is highly compatible with the current research on efficient sense-storage computing systems. In particular, the MXene self-powered vertical friction transistor developed by Liu et al., by structurally fusing a triboelectric nanogenerator (TENG) with the transistor, not only realizes the breakthrough application of high-precision multimodal emotion recognition but also constructs a new paradigm for tactile sensing systems [95]. This simplified processing mechanism of data conversion, transmission, storage, and operations eliminates the loss of cross-module data conversion and redundant transmission in traditional architectures and provides a verifiable technological prototype for realizing the full-process fusion of sense-storage-computing in the field of in-sensor computing.

#### Chemical Perception

**Fig. 17:**
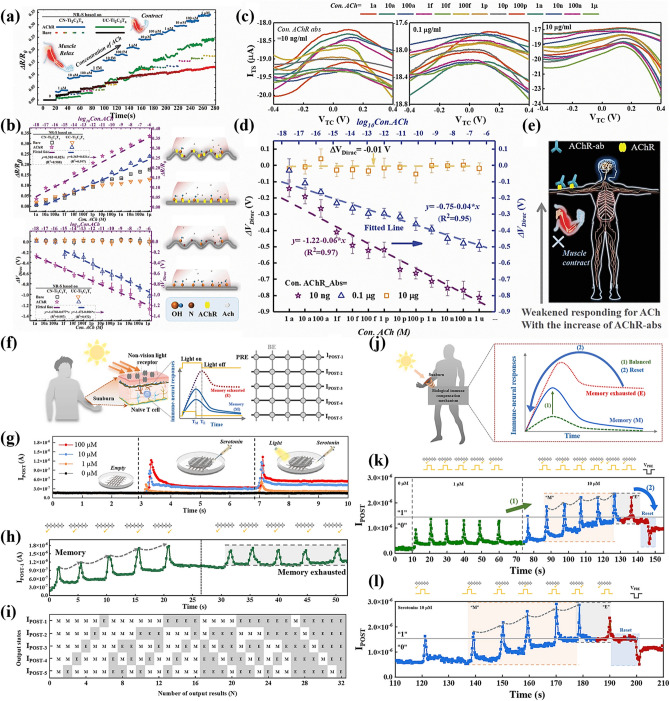
**a** Change ratios of transient resistance (∆R/R_0_) with increasing concentrations of ACh (Con. ACh). **b** Linearly fitted curves of ∆R/R_0_ versus Con. ACh for the AChR modified devices, in contrast to the scattered data of the bare ones, and the changed voltages of Dirac points (∆V_Dirac_) versus Con. ACh. **c** Shifted transfer characteristic curves with increasing Con. ACh, measured by the CN-Ti_3_C_2_T_x_-based NR-S after incubation with AChR-ab solutions at concentrations of 10 ng mL^−1^, 0.1 µg mL^−1^, and 10 µg mL^−1^. **d** Shifted voltages of Dirac points (∆V_Dirac_) versus Con. ACh curves. **e** Schematic diagram for the damaged neuron. Reproduced with permission [30]. Copyright 2021, Wiley–VCH GmbH. **f** Schematic diagram of fibrous synapse array and skin immune T cell responses to sunshine. When the skin is exposed to sunlight, with increasing sunshine duration from TM to TE, T cells lose their memory function and reach the exhausted state. **g** I_POST_ of one fibrous synapse when the serotonin concentrations are increased from 0 to 100 µM and the light pulses are applied. **h** I_POST_ of five fibrous synapses in a line after being illuminated by light pulses, with a serotonin concentration of 10 μM. **i** Output states of I_POST-i_ (*i* = 1, 2, 3, 4, 5) of the fibrous synapses array when illuminated by different methods under working condition of VPRE = 1 V, 10 µM mL^−1^ serotonin and 10 mW cm^−2^ light pulse. **j** Schematic diagram of the biological immune compensation process in response to light. **k** Simulation of the balanced immune compensation process in which the spike signals of I_POST_ are improved by increasing the serotonin concentration from 1 to 10 µM when V_PRE_ = 1 V, and the exhausted state (“E”) signal is RESET by the negative pulse of V_PRE_ (−1 V). **l** After the RESET operation, the continuously simulated immune-neural responses (I_POST_) are conducted under the same working condition in **b** (V_PRE_ = 1 V and 10 µM serotonin). Meanwhile, the E state response can also be RESET by the negative pulse of V_PRE_ (− 1 V), simulating the reset immune compensation. Reproduced with permission [28]. Copyright 2024, Elsevier Ltd [29]

The above studies simulated biological neural response processes by using the conductance modulation behavior of artificial synaptic devices and constructed bionic neuromorphic systems close to near-sensor computing and in-sensor computing. However, the real biological neural system is not solely reliant on neuroelectric signals; it is also modulated by the biochemical environment within the organism [96, 97]. To accurately simulate the real biological response process, it is imperative that artificial synaptic devices be capable of being modulated by neurotransmitters and other chemicals, which remains a challenge for traditional artificial synaptic devices. Therefore, the development of a biochemically regulated artificial synaptic device could significantly advance biomimetic applications in neuromorphic systems. Wang et al. proposed a neurotransmitter receptor-mediated three-terminal artificial synapse (NR-S) device [30]. They constructed the MXene-PBS solid–liquid interface as the third-terminal functional electrode by extending the MXene-Ti_3_C_2_T_x_ functional layer of the memristor. Subsequently, after modifying the neurotransmitter receptor (AChR) at the third-terminal functional electrode interface, the device achieved neurotransmitter (ACh) modulated biomimetic neural properties, as shown in Fig. 17a. Specifically, the simulation of synaptic plasticity behaviors was accomplished by varying the ACh concentration. The limit of detection (LOD) of the device was increased to 1 aM by N-doped crumpled MXene nanosheets (CN-Ti_3_C_2_T_x_), as shown in Fig. 17b. Also, this work simulated the neuronal damage response induced by pathogenic autoantibodies using CN-Ti_3_C_2_T_x_-based NR-S devices. NR-S devices modified with AChR were immersed in different concentrations of AChR-Ab solution (i.e., Con. AChR-Ab = 10 ng mL^−1^, 0.1 µg mL^−1^, and 10 µg mL^−1^) for modification. Subsequently, the response of NR-S devices modified with these different concentrations of AChR-Ab to ACh solutions was evaluated using transfer characteristic curves. As shown in Fig. 17c, when the concentration of AChR-Ab was relatively low (10 ng mL^−1^, 0.1 µg mL^−1^), the Dirac point shifted to higher values with increasing ACh concentration. When the concentration of AChR-Ab was increased to 10 µg mL^−1^, the Dirac point did not change. The subsequent changes in the Dirac points at the three AChR concentrations are depicted in Fig. 17d. The neural response of the NR-S devices to ACh almost disappeared when the NR-S devices were treated with high concentrations of AChR-Ab (10 µg mL^−1^). These phenomena are consistent with the neurochemical studies on myasthenia gravis in Fig. 17e, where the greater damage to AChR results in a lower neuronal response to ACh. This result suggests that this NR-S device could be useful in aiding the early diagnosis of myasthenia gravis [98].

Furthermore, this research team prepared a fibrous artificial synaptic array with Ag/MXene/MoS_2_/Pt structure and evaluated the possibility of synaptic devices mimicking chemical modulation in a multi-biological clock system [29]. The immune-neural responses in both memory and memory exhaustion, shown in Fig. 17f, were developed as an example of dual chemical and optical modulation of the immune-neural responses. When the V_PRE_ was 1 V (> V_TH_), I_POST_ was measured after different serotonin and light stimuli, as shown in Fig. 17g. When no serotonin stimulation was applied, the I_POST_ values were stable and remained constant. After serotonin stimulation was applied, the I_POST_ curves showed an obvious jumping behavior, and the jumping peaks of the I_POST_ curves (depicted in orange, blue, and red) were all enhanced as the serotonin concentration increased from 1 to 100 μM. This result demonstrated that the proposed fibrous synapses have the ability to modulate synaptic plasticity in response to serotonin. At 7 s after 10 mW cm^−2^ light stimulation, the I_POST_ curve jumped again and dropped to a new level, implying that nonvisual light stimulation (one of the elements in the peripheral clock) can co-modulate synaptic currents with the main clock element (serotonin). The output current of one row in a 5 × 5 array of fibrous synapses (I_POST−1_) was subsequently measured and is plotted in Fig. 17h. It can be observed that the current value of I_POST−1_ gradually increases with each light pulse stimulation, indicating that external light stimulation can be memorized by the synaptic device. The memorization effect is enhanced with the increasing number of stimulations. However, this memory enhancement ceases after 5 light pulses, indicating that the synaptic device reaches an exhaustion state. Finally, the I_POST-i_ (i = 1, 2, 3, 4, 5) for 5 × 5 synaptic arrays, which were irradiated with different numbers of light pulses, were collated. The 32 different outputs are shown in Fig. 17i. It is suggested that the number of exhaustion states (“E”) increases as more light pulses are applied, analogous to skin burns from overexposure to sunlight. Based on the skin immune-neural response to serotonin and light, fibrous synaptic arrays were used to simulate the process of skin immune compensation to light, as shown in Fig. 17j. Serotonin concentration served as the master clock signal, while the number of light pulses simulated skin exposure to sunlight [99]. The spiking signal of I_POST_ was employed to mimic an immune-neural response, and the experiment was conducted at V_PRE_ = 1 V, as depicted in Fig. 17k, l. During the 0 and 10 s, when neither serotonin nor external light was applied, I_POST_ remained approximately zero. Upon applying a low concentration of serotonin (1 µM) along with light pulses from 11 to 70 s, I_POST_ exhibits six low intensity spike signals, resembling a weak immune-neural response. I_POST_ levels were significantly elevated after the application of 10 µM serotonin, akin to the organism’s balanced immune compensation [100]. The sustained I_POST_ signal mimics the immune-neural response observed in the exhausted state of memory T cells during sunburn, which occurs after exposure to an excessive light pulse. In this study, a reverse bias voltage V_PRE_ = −1 V was utilized to simulate the RESET immune compensation of stem cells. It was observed that, upon applying the reverse V_PRE_, the I_POST_ spike signal was inverted, and the IPOST curve reverted to its initial low level. Upon continuing to simulate the immune-neural response after RESET, it was evident that the I_POST_ spike signal gradually intensified to a high level as the number of light pulses increased, and both memory and memory exhaustion behaviors could be replicated. In summary, it can be confirmed that MXene-Ti_3_C_2_T_x_-based fibrous synaptic arrays can simulate immune compensation responses that are co-modulated by the master clock and the peripheral clock. This finding suggests that the proposed device can provide a possible tool for the application of neuromorphic devices in immune learning.

#### Optic Perception

**Fig. 18:**
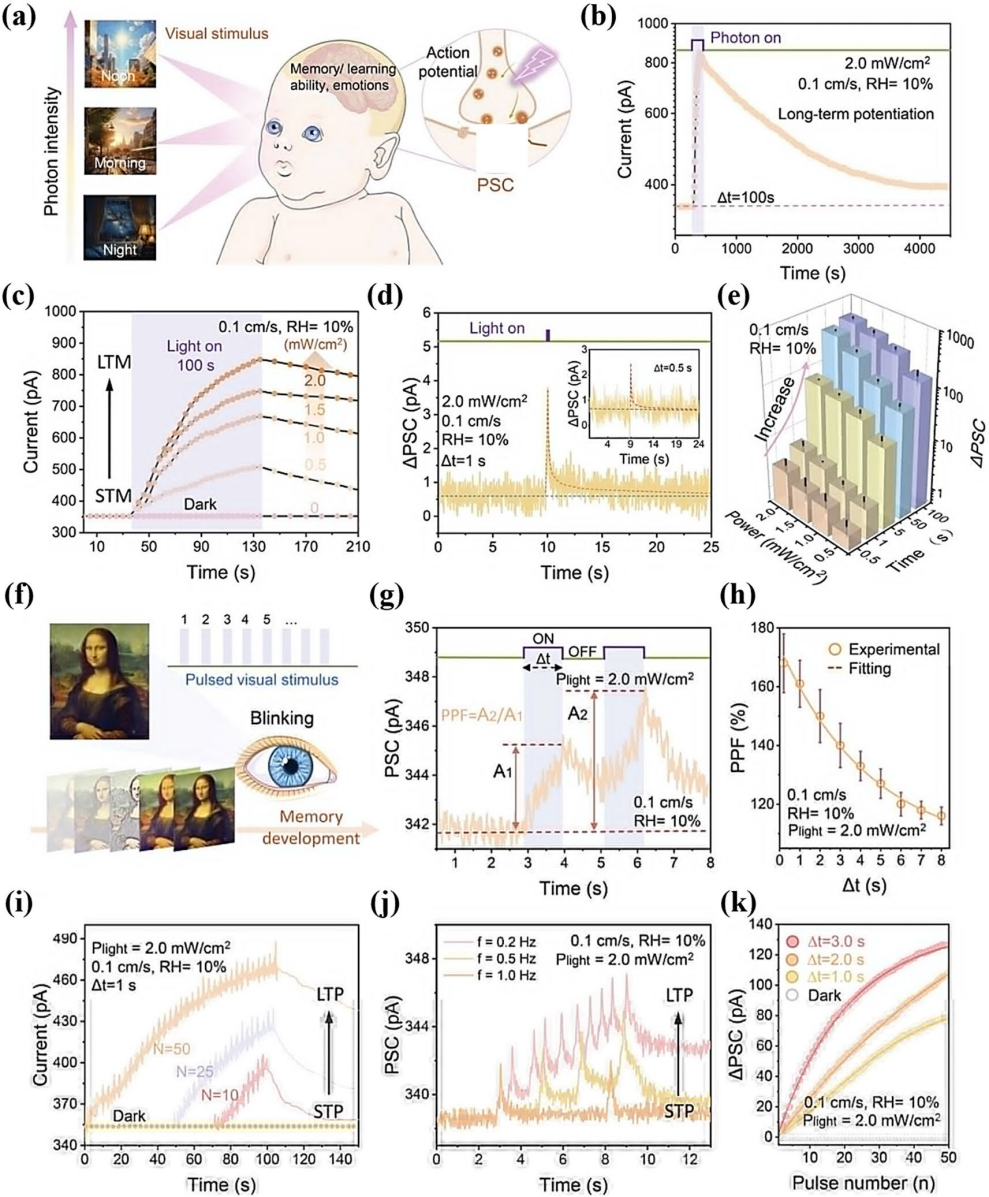
Photoexcitation characteristics of the VRSOM. **a** Schematic diagram of the formation of neural excitability in the human eye with various light-intensity environments. **b** Typical current evolution process of the VRSOM under light stimulation (405 nm, 2.0 mW cm^−^.^2^). **c** Current response is triggered by different light intensities, and switching phenomena from STM to LTM. **d** PSC with single-pulse light stimulation. **e** Current changes of the VRSOM under light stimuli with various intensities and pulse widths. **f** Schematic diagram of human brain neuroexcitatory state in the case of pulsed visual signal input. **g** Typical photoexcitation characteristic of the VRSOM under a continuous light pulse pair with 1 s interval. **h** Variation of the PPF index with the interval of light pulse pairs. **i**, **j** STM to LTM transition induced by increased pulse number and various frequencies, respectively. **k** PSC changes under different types of paired pulses. Reproduced with permission [93]. Copyright 2024, Wiley–VCH GmbH [101]

To promote the application of multimodal in-sensor computing technology, many researchers have devoted themselves to the exploration of optical signal perception and memory technology. They are focused on researching how to realize the efficient transmission and processing of optical signals, to open up new ways for the fusion and analysis of multimodal information. Tan et al. reported an artificial visual-respiratory synapse with monolayer oxidized Ti_3_C_2_T_x_ MXene (VRSOM) and achieved photo-modulated synaptic plasticity behavior and biomimetic features [101]. Figure 18a illustrates a schematic diagram of visually modulated synaptic behavior, in which the human eye responds to visual stimuli (intensity, timing, and pattern), similar to the effects of EPSC on mood, memory, and learning ability. The optical synaptic plasticity of VRSOM was first explored. As shown in Fig. 18b, the VRSOM exhibited LTP under continuous illumination (100 s.) for more than 4000 s. With the increase in light intensity, the photocurrent of the device gradually increased, and the PSC was transformed from STP to LTP as shown in Fig. 18c. Figure 18d reveals that a 1 s light pulse can cause a current change of 4.0 pA. To investigate its photoexcitation properties in detail, the neural response to light stimulation of different durations and intensities was measured, as shown in Fig. 18e, which indicates that the current increases with increasing power and pulse duration. Figure 18f illustrates the process of memory formation for impulsive and repetitive stimuli during visual interactions, where visual signals are typically input intermittently. This neuromorphic behavior is simulated in Fig. 18g by pairs of light pulses with an interval and width of 1 s inducing photoexcitation currents, and it was found that higher excitation currents were observed with the second light pulse. The simulation of PPF was subsequently completed by photo-modulation as shown in Fig. 18h.

To investigate the transition process from STM to LTM, the VRSOM device was stimulated using different numbers of pulses (N = 10, 25, 50). In Fig. 18i, the PSC exhibits STM behavior with a small number of stimuli (N = 10). With the increasing number of pulses, the current level increased, indicating that the device achieved the transition from STM to LTM. In addition, light pulses of different frequencies also induced changes in the conductivity of the VRSOM, as shown in Fig. 18j. As the pulse frequency increases, the PSC increases but the decay rate slows down. Finally, the PSC for the same number of pulses (N = 50) at different interval times is shown in Fig. 18k, demonstrating the correlation between PPF and interval time. VRSOM contributes to the development of interactive photonic synapses and artificial systems, facilitating innovative ways of neural network research and in-sensor computing. Lee et al. developed a flexible optoelectronic artificial synaptic device based on MXene-Ti_3_C_2_T_x_, which was integrated on a polyimide substrate by solution processing technology and exhibited a broad-band photoresponsive and memory effect from the visible to the near infrared. The study successfully modeled the core features of synaptic plasticity, including EPSCs, PPF, STM, and LTM. The technique was applied to two types of neuromorphic computing scenarios: (1) pattern recognition in artificial neural networks, with 90% accuracy in deformation-resistant recognition, and (2) construction of a Pavlovian conditioned reflex model, which verified the bionic implementation of associative learning behaviors. This breakthrough provides an important technological path for the development of novel neuromorphic optoelectronic systems [102].

**Fig. 19:**
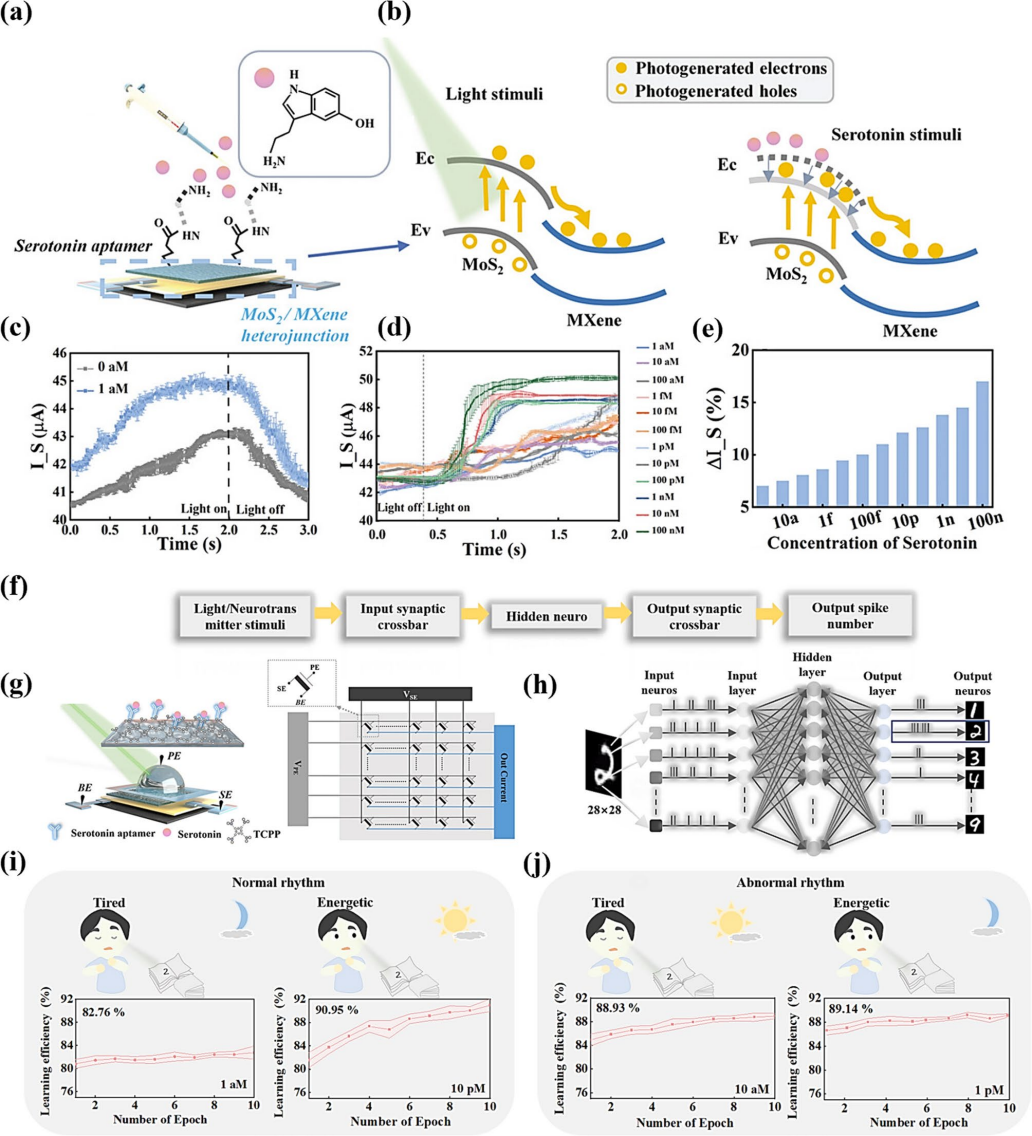
**a** Schematic diagram of the OANS device under the dual modulation by light and serotonin. **b** I_S is modulated by increasing serotonin and light excited band bending. **c** On–off light triggered dynamic I_S curves before and after cross-linking with 1 aM serotonin, V_PE_ = −2 V, V_SE_ = 0.1 V and light intensity was 5 mW cm^−2^. **d** I_S dynamic curves under on–off light (5 mW cm^−2^), with increasing serotonin concentration (1 aMM−100 nMM), V_PE_ = − 2 V, V_SE_ = 0.1 V. The error bars represent the standard deviation of five randomly selected individual OANS devices. **e** ∆I_S triggered by on–off light, with increasing serotonin concentration (1 aM−100 nM). **f** Flowchart of the simulation process. **g** Schematic of the hardware implementation of the light and neurotransmitter dual stimulated neural network by using the proposed optic nerve synaptic device at each intersection. **h** Schematic of the SNN for MNIST classification. It contains 784 input neurons (OANS device), 100 hidden neurons, and 10 output neurons. **i** Two learning efficiency results related to the normal circadian rhythm behaviors: tired at night and energetic during the day. **j** Two learning efficiency results related to the abnormal circadian rhythm behaviors, tired during the day and energetic at night. The error bars represent the standard deviation of five randomly selected individual OANS devices

Neurological studies have shown that human learning and memory are characterized by circadian rhythms, and the optic afferent neural system (OANS) plays a key role in maintaining circadian rhythms [103, 104]. Biomimetic simulation of the OANS function contributes to the multimodal development of in-sensor computing applications. Wang et al. reported an OANS device based on MXene/MoS_2_ heterojunction and used it for simulation experiments to realize the learnability of circadian rhythms [105]. The experimental scheme is shown in Fig. 19a. The device can convert optical signals into synaptic currents and can be modulated by V_PE_. If a biochemically modified interface is embedded on the MoS_2_ surface, biochemical tuning of the effective bias voltage on the heterojunction can be achieved, leading to energy band bending (Fig. 19b). To demonstrate this design, a 5-hydroxytryptamine (5-HT)-specific aptamer was crosslinked on a TCPP-modified MoS_2_/MXene heterojunction. The effects of different concentrations of neurotransmitters on I_S were examined under controlled V_PE_, V_SE_, and light intensity. It was found by the dynamic I_S curve in Fig. 19c that the same light triggered an upward shift of I_S when the 5-HT concentration was increased from 0 to 1 aM, which was attributed to the bending of the heterojunction bands induced by the captured 5-HT molecules. The resistive state of the OANS device was shown to be modulated by neurotransmitter concentration in Fig. 19d, and the rate of current change is summarized in Fig. 19e. Subsequent experiments to simulate the learnability of circadian rhythms were performed using the developed bionic OANS device, as shown in the flowchart in Fig. 19f. As shown in Fig. 19g, the OANS device was used to sense external stimuli and was located at the cross point of the neuromorphic array. The collected I_S data are input to a three-layer spiking neural network (SNN) and filtered through a brain-like analysis to output a “recognized” digit (Fig. 19h).

The learning efficiency of the proposed OANS is evaluated by using the MNIST handwritten digit classification results, i.e., the learning efficiency is evaluated by the mean and standard deviation of the recognition accuracies of five randomly selected OANS devices. Figure 19i, j shows the schematic and test results of the simulated circadian rhythm learning ability. In Fig. 19i, boys with normal circadian rhythms would be tired and less efficient in learning (82.76%) at night, which was simulated by the absence of light and 1 aM serotonin. Boys with normal circadian rhythms would learn more efficiently during the day (90.95%), which was simulated by applying 5 mW cm^−2^ light intensity and 10 pM serotonins.

As shown in Fig. 19j, boys with abnormal rhythms get tired during the day (88.93% learning efficiency), which was simulated by the absence of light and 10 aM serotonin. In contrast, boys with abnormal rhythms have relatively high learning efficiency at night (at 89.14%), which was simulated with 5 mW cm^−2^ light intensity and 1 pM serotonin applied. The simulation results are consistent with neurobiological studies. Therefore, this OANS device enables the simulation of circadian rhythmic learning behaviors. Wu et al. developed a bilayer asymmetric ionic hydrogel device with self-powered neuromorphic sensing that can simultaneously combine tactile and visual perception. The upper layer of the device is uniformly doped with MXene nanosheets for photothermal conversion. The mechanism combines piezoelectric and ionic thermal diffusion effects to generate ionic potentials that mimic the memory effect of postsynaptic potentials (PSP) utilizing relaxation properties. The device is able to reproduce short/long-term synaptic plasticity under pressure and light stimulation, realizing multimodal sensing fusion in a single device. In addition, its multimodal sensing capability significantly improves the grasping control precision and environmental adaptability of the robotic arm, offering a new idea for the development of intelligent bionic systems [106].

### Integrated Simulation Computing

**Fig. 20:**
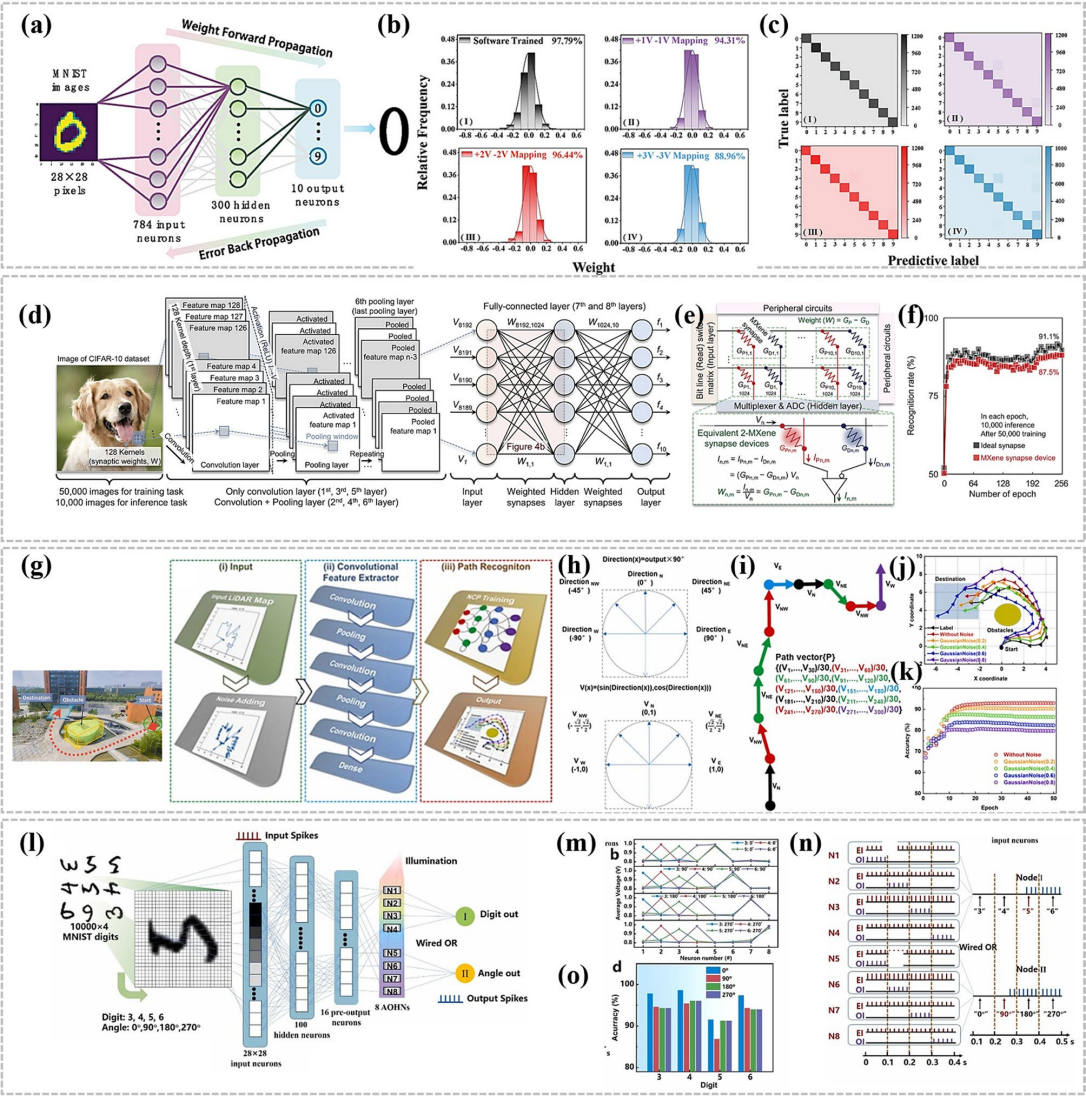
**a** Schematic of a three-layer fully connected ANN with 784 input neurons, 300 hidden layer neurons, and 10 output neurons. **b** Weight distribution, recognition accuracy. **c** Confusion matrix of software-trained and spike amplitude modulation. Reproduced with permission [23]. Copyright 2023 Wiley–VCH GmbH [24]. **d** CIFAR−10 dataset and convolution neural network composed of the MXene synapse devices. **e** Conceptual circuit diagram of neural network comprising the MXene synapse devices corresponding to the fully connected layer of CNN (top), and conceptual circuit diagram that corresponds to synapse weight composed of two equivalent MXene synapse devices (bottom). **f** Recognition rate with respect to the number of epochs for the CIFAR−10 dataset, where ideal and MXene artificial synapses were employed. Reproduced with permission [41]. Copyright 2021 Wiley–VCH GmbH. **g** Schematic illustration of the path recognition task, and the structure of the NLP-based neural network computing system for path recognition task. **h** Vector maps and coordinates for directions, and the scalar of each vector set to fit a standard distance. **i** Configuration of a pathway vector (P), including thirty directional vectors. **j** Path recognition results with and w/o various standard deviations of Gaussian noises. **k** Recognition accuracy of path recognition as functions of learning epochs under different levels of Gaussian noises. Reproduced with permission [40]. Copyright 2023, Elsevier Ltd. **l** Network of SNN to recognize the digit patterns and rotation angles of 4 × 4 patterns, where the recognition process includes pixel encoding, weight multiplication and addition, signal accumulation, and spatiotemporal integration. **m** Average input signals to 8 AOHNs in the 16 input patterns recognition process. **n** Spatiotemporal integration of EI and OI, which can recognize the digit patterns and rotation angles according to the fire time of two nodes. **o** Recognition accuracy of 16 input patterns. Reproduced with permission [97]. Copyright 2022 Elsevier Ltd [107]

Currently, memristors can be applied to large-scale neuromorphic computation due to their nonvolatile and highly integrated features, and thus, many works use memristor-based neural network simulations to verify their feasibility in hardware design. Huang et al. tested the conductance modulation results of flexible artificial synapse arrays based on TiO_x_/Ti_3_C_2_T_x_ films using different pulse voltages [24]. The fully connected artificial neural network (ANN) based on artificial synapses, for recognizing and classifying handwritten digits, was subsequently simulated on the Modified National Institute of Standards and Technology (MNIST) database. The fully connected neural network contains 784 input neurons corresponding to an input MNIST image of size 28 × 28 pixels, 300 hidden layer neurons, and 10 output neurons corresponding to 10 classes of digits (0–9), as shown in Fig. 20a.

Figure 20b shows the probability and accuracy plots of weight distribution under different voltage pulse modulations. It can be found that the highest recognition accuracy of the neural network under 2.0 V pulse voltage modulation is 96.44%, which is close to the recognition accuracy of the optimal weights in software training (97.79%). Finally, the confusion matrix comparing predicted labels with real labels is displayed (Fig. 20c). The TiO_x_/Ti_3_C_2_T_x_-based memristor demonstrates high recognition accuracy in recognizing handwritten digits on the MNIST database through simulations of fully connected neural networks, indicating its reliability for further construction of high-density artificial synapse arrays and large-scale integration of neuromorphic computational circuits in the future. An artificial synaptic device based on 2D MXene-Ti_3_C_2_T_x_ nanosheets was prepared by Ju et al. [41]. A convolutional neural network (CNN) was constructed based on the realization of synaptic plasticity behaviors such as EPSC/IPSC. The left side of Fig. 20d shows the 50,000-image CIFAR−10 dataset used for training and the 10,000-image CIFAR−10 dataset used for validation. The right side of Fig. 20d shows the eight-layer CNN including convolutional, pooling, and fully connected layers used for training. As shown in Fig. 20e, the synaptic weight was defined as the difference between the conductance values of two equivalent MXene synapses (W = G_P_-G_D_). The weights were increased (W↑ = G_P_↑-G_D_↑) or decreased (W↓ = G_P_↓-G_D_↑) by increasing and decreasing the conductance values of the MXene devices. The neural network recognition rates for the ideal condition and the real MXene synapses after training using CNN and CIFAR−10 datasets are shown in Fig. 20f. After 12 training cycles, the MXene synapse-based neural network recognition improves to 82% and reaches a maximum accuracy (87.5%) after 244 training sessions, which is close to the recognition rate of neural networks with ideal conditions (91.1%). Cao et al. presented an in-sensor computing system based on the GeOx-MXene nanosheets visual artificial synaptic TFTs (GMXST) and applied to a path recognition task, as shown on the left side of Fig. 20g [40]. The right side of Fig. 20g shows the structure of neural circuit policies (NCP)-based neural network computing system for this path recognition task. Figure 20h depicts the method of constructing a path from the NCP output with the help of eight direction vectors. The configuration of the path vector (P) consists of thirty direction vectors, as shown in Fig. 20i. Gaussian noise with standard deviations of 0.2, 0.4, 0.6, and 0.8 is added to the input data, and the results of path recognition are shown in Fig. 20j, k. The high noise tolerance of the NCP-based path recognition system is demonstrated by over 80% accuracy with 0.8 standard deviation Gaussian noise. This further demonstrates the potential of MXene-based neuromorphic devices for in-sensor computing applications. An artificial neural device with ITO/PVA: MXene-Ag NPs/Ag structure was constructed by Yu et al. An SNN with 28 × 28 inputs was composed using this device and neurons, as shown in Fig. 20l [107]. 10,000 numbers with four degrees of rotation (0°, 90°, 180° or 270°) were selected from the MNIST database. Due to the rotational asymmetry of these digits, the selected digits were limited to 3, 4, 5, and 6. The SNN was then constructed with 784 input neurons, 100 hidden neurons, and 16 output neurons, with neurons 1–4 regarded as the digit outputs and neurons 5–8 as the angle outputs, respectively. After 100 iterations of training, the average voltage of 8 neurons is shown in Fig. 20m. Figure 20n demonstrates the simplified process of the neural network. In the simulation, the simplified recognition scheme exhibits higher accuracy as shown in Fig. 18o. After 50 training cycles, an average accuracy of 93.6% can be achieved, which shows that the MXene-based neuromorphic device can provide support for multitasking neural networks and in-sensor computing applications.

### Integrated Hardware Computing

**Fig. 21:**
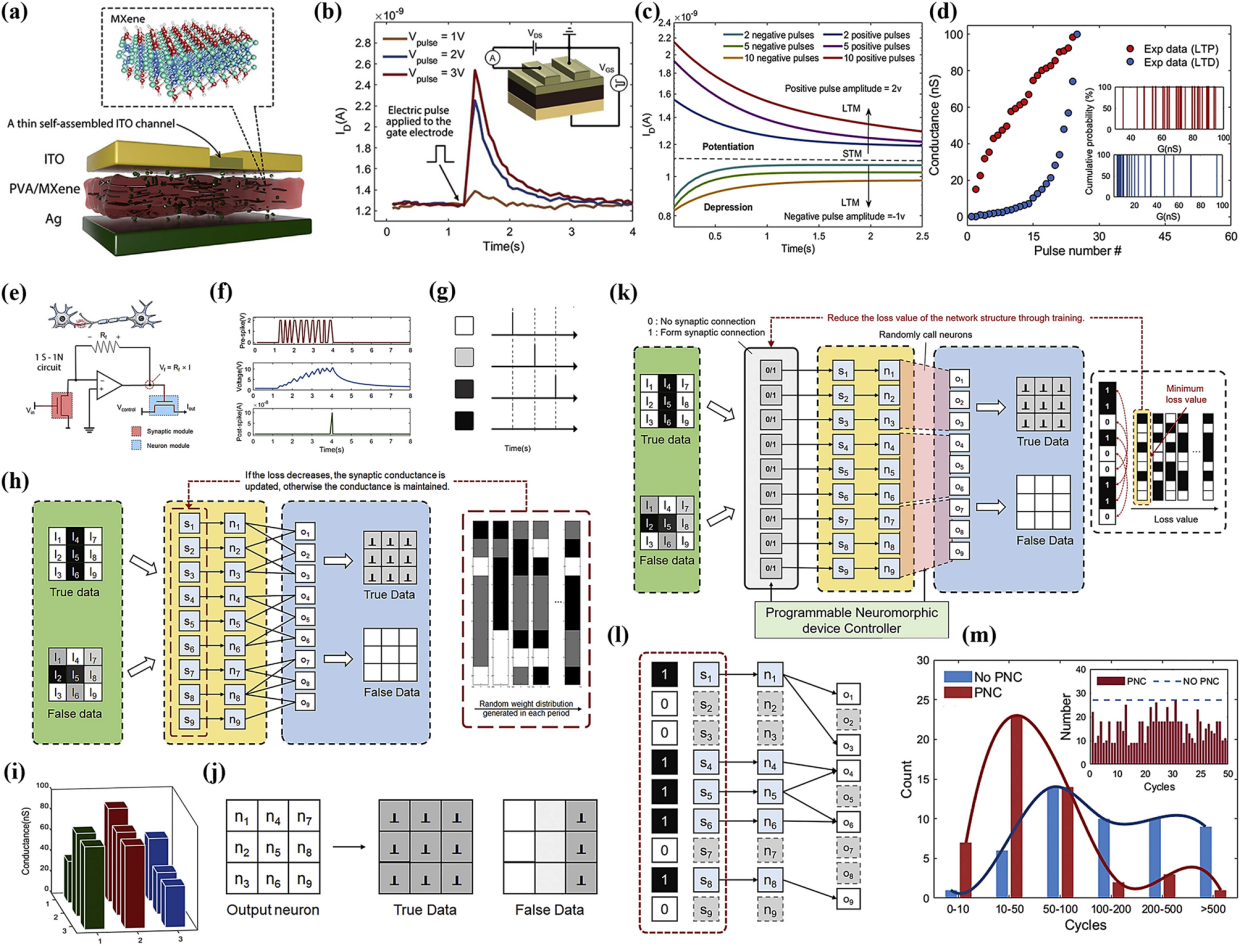
**a** Schematic of the SNST and the molecular structure of MXene. **b** EPSC triggered by a presynaptic pulse (1, 2, and 3 V; T_width_ is 60 ms) as a function of time at V_DS_ = 3 V. **c** Decay processes after different numbers of pulses (2, 5, and 10 positive/negative pulses, positive input voltage = 2 V, negative input voltage = − 1 V, T_width_ is 60 ms, and T_interval_ is 0.3 s). **d** Relationship between the conductance of the SNST and the number of pulses. (T_width_ is 60 ms, V_input_ is ± 1 V, and T_interval_ is 0.3 s.) **e** Schematic of the 1S−1N circuit. **f** The first row is the input of 1S−1N circuit, the second row is the output of the amplifier as the input of the neuron, and the last row is the output of the 1S−1N circuit. **g** The first spike emitting time coding of gray information. **h** Structure of a neuromorphic hardware network based on the SNST, which consists of an input layer (the green area on the left), hidden layer (the orange area in the middle), and output layer (the blue area on the right). **i** Synaptic weight distribution of the trained hardware network. **j** Output of the trained hardware network and the output neurons will burst when the input image is a real image. **k** Structure of neuromorphic hardware network based on the SNST with the PNC (the cyan area at the bottom). **l** Topology of the hardware network with the PNC after training. **m** Comparison chart of the hardware network with and without the PNC. Reproduced with permission [23]. Copyright 2022 Elsevier Inc.

Memristor-based neuromorphic hardware networks are an order of magnitude or more energy-efficient than traditional graphics processors (GPUs) when dealing with tasks such as CNNs [108, 109]. This means that the memristor-based neuromorphic network can perform computations with lower power consumption for the same computational task. Memristor crossover arrays support parallel matrix multiply–add operations, which enables memristor-based neuromorphic hardware networks to handle multiple computational tasks simultaneously, thus accelerating the training process of neural networks. Zhang et al. proposed a 2D MXene-Ti_3_C_2_T_x_-based switchable neuronal synaptic transistor (SNST) and programmed to realize synaptic and neuronal functions [23]. Figure 21a shows the Ag/PVA/MXene-Ti_3_C_2_T_x_/ITO-based SNST device with bottom gate and top contact electrodes. The test in Fig. 21b reveals a slower current decay at high amplitude voltage pulses, which is similar to the EPSC. When voltage pulses are applied to the gate of the SNST, the response of the ID is similar to the transition from STM to LTM. As shown in Fig. 21c, the ID can change with the increasing number of positive and negative pulses. The relationship between the number of pulses and the drain–source conductance is shown in Fig. 21d, indicating that the maximum conductance (G_max_) of the indicated SNST is 95 nS, and the minimum conductance (G_min_) is 4 nS. The cumulative probability distribution of the 25 independent conductance states of the different SNSTs is also shown in Fig. 21d, which shows a good uniformity.

Figure 21e shows a 1S−1N circuit comprising a synaptic SNST device, a neuronal SNST device and other electronic components. Using this 1S−1N circuit and the first pulse emission time coding method, grayscale information encoding is achieved. As shown in Fig. 21e, in the 1S−1N circuit, the grayscale information is expressed in terms of the number of pulses and used as the input to the synapse, and the postsynaptic current is amplified by an amplifier as the input to the neuron. The timing of the first output spike of the 1S−1N circuit represents the grayscale information, as shown in Fig. 21f, where the first row is the input of the 1S−1N line, the second row is the output of the amplifier that serves as the input to the neuron, and the last row is the output of the 1S−1N line. Different grayscale information can be encoded by spiking signals with different firing times, as shown in Fig. 21g.

Using this 1S−1N circuit, a neuromorphic hardware network simulation was conducted to verify the authenticity of the input data, as shown in Fig. 21h. If the input data are the same as the real data, the output neurons will all fire spikes; otherwise, only some neurons will fire. After several training cycles, the synaptic weights of the hidden layer are redistributed to obtain an ideal hardware network. The synaptic weight distribution of the hidden layer of the hardware network after training is shown in Fig. 21i. As shown in Fig. 21j, the task of distinguishing between true and false input images is successfully realized. Based on this hardware network, a programmable neuromorphic device controller (PNC) was added to change the connection modes of the SNSTs. The PNC can be used to control nine 1S−1N circuits to be switched on (Logic 1) or off (Logic 0), as shown in Fig. 21k. The topology of the PNC hardware network, as depicted in Fig. 21l, reveals that only 10 SNSTs are necessary to fulfill the initial network function that would otherwise require 27 neuromorphic devices, thereby demonstrating the efficiency of the PNC hardware network. Figure 21m illustrates the number of training cycles required to reach the optimal state, highlighting a significant reduction in the number of training cycles needed for the PNC-based hardware network.

## Summary and Outlook

In summary, this review focuses on MXene-Ti_3_C_2_T_x_, the star member of MXene family, and provides a comprehensive overview of its latest research results and developments in the field of neuromorphic devices. Within this paper, we first discuss in depth the key strategies to enhance the performance of MXene-Ti_3_C_2_T_x_ neuromorphic devices, summarizing three mainstream material optimization paths: The first is to adjust the interfacial properties of the materials through surface modification techniques to enhance their biomimetic neural properties. The second is to endow the devices with richer functional properties through nanoparticle doping. The third is to optimize the charge transport and storage mechanism through the dielectric layer coupling method to further enhance the performance. Notably, MXene-Ti_3_C_2_T_x_ demonstrates unique advantages in the integration of sensing, storage, and computing, thereby enabling in-sensor and near-sensor computing paradigms that circumvent the von Neumann bottleneck inherent in traditional CMOS architectures. In contrast to CMOS devices, which are constrained by the physical separation of sensing, memory, and processing units, MXene-based systems achieve parallel multimodal signal processing with ultra-low power consumption. Subsequently, the paper systematically analyzes several core physical mechanisms in the MXene-Ti_3_C_2_T_x_ neuromorphic device, including ECM, VCM, and pure electron effect. The in-depth analysis of the physical mechanisms provides a solid theoretical foundation for the application of MXene-Ti_3_C_2_T_x_ in neuromorphic computing. Finally, this paper highlights the innovative applications of MXene-Ti_3_C_2_T_x_ neuromorphic devices in the cutting-edge computational paradigm, that is, near-sensor computing and in-sensor computing. By detailing the in-device simulation strategy based on electrical signals and the out-device simulation strategy based on multimodal signals, the information dimension of neuromorphic computation is further broadened. These integrated capabilities starkly contrast with conventional CMOS systems that require complex peripheral circuits for analog-to-digital conversion and data shuttling between discrete components, resulting in massive energy overhead and latency. The exploration of MXene-Ti_3_C_2_T_x_ neuromorphic devices in both integrated simulation computing and integrated hardware computation is also presented, demonstrating the great potential of MXene-Ti_3_C_2_T_x_ devices in building highly integrated, low-power, high-performance neuromorphic systems. Based on the above research, this review is carefully compiled in Table 1, aiming to comprehensively and systematically integrate almost all research results involving MXene-Ti_3_C_2_T_x_ neuromorphic devices since 2019. The table exhaustively lists diverse structural designs of MXene-Ti_3_C_2_T_x_ devices and provides an in-depth analysis of their electrical performance characteristics, including, but not limited to the switching voltage, endurance, and power consumption. It also summarizes switching mechanisms and the biomimetic and chemical properties inspired by the biological nervous system are also summarized, which are criteria for evaluating neuromorphic devices performance. Therefore, Table 1 serves as both a comprehensive review of recent MXene-Ti_3_C_2_T_x_ neuromorphic devices and a detailed reference guide for researchers in materials and neuromorphic computing, promoting the rapid development of neuromorphic technology.

**Table 1:** MXene-T_i3_C_2_T_x_-based neuromorphic device

Deice structure	Switching type	SET Voltage (V)	RESET Voltage (V)	On/Off ratio	0	Endurance (N)	Power consumption (J)	SET power (W)	Resistance switching mechanism	Biological characteristics	Refs
Al/Ti_3_C_2_T_x_/Pt	Bipolar switching	4.5	−3.8	10^3^	10^5^	10^6^	/	4.5 × 10^–6^	Electron tunneling	PPF/STDP/STM/LTM	[12]
Pt/Ti_3_C_2_T_x_/ITO/Si	Bipolar switching	1.95	−2.04	10^2^	10^4^	10^3^	/	2 × 10^–3^	VCM	LTD/LTP	[39]
Ag/Ti_3_C_2_T_x_-TiO_2_/Pt	Bipolar switching	3.3	−5	10^6^	/	2 × 10^2^	18.82 × 10^–9^	2.64 × 10^–9^	/	PPF/STP/LTP/LTD	[110]
Al/Ti_3_C_2_T_x_-TiO_2_ NF/Pt	Bipolar switching	0.68	0.53	2.3 × 10^3^	10^5^	10^4^	/	1.36 × 10^–4^	VCM	LTP/LTD/STDP	[111]
Ag/TiOx/Ti3C2Tx/Au	Bipolar switching	1.6	−1.8	10^2^	10^3^	10^2^	/	1.6 × 10^–5^	ECM	LTP/LTD/STP/PPF	[24]
Ag/Ti_3_C_2_T_x_:Ti0_2_/Pt	Bipolar switching	1.33	−0.94	10^4^	10^4^	10^3^	27 × 10^–9^	1.33 × 10^–5^	ECM	STDP/LTP/LTD/PPF/EPSC	[38]
Cu/SnS/Ti_3_C_2_T_x_/Cu/Si	Bipolar switching	1.5	/	22	10^3^	10^3^	/	/	Charge trapping	/	[112]
Al/ZTO/TiO_x_/Ti_3_C_2_T_x_ SiO_2_/Al	Unipolar switching	/	/	/	/	/	100 × 10^–15^	/	Charge trapping	IPSC/EPSC/PPF/STM/LTM/LTP/LTD	[113]
PEI/Ti_3_C_2_T_x_/PSS	Bipolar switching	1.5	−2	/	/	/	/	/	VCM	/	[114]
Cu/Ti_3_C_2_T_x_/Cu/SiO_2_/Si	Bipolar switching	0.68	−0.61	10^3^	/	/	2 × 10^–8^	2 × 10^–6^	ECM	SRDP/STDP/LTP/STP/STD/PPF	[115]

Ag/Ti_3_C_2_T_x_-CB/ITO	Bipolar switching	0.9	−1.1	10^2^	10^4^	/	/	/	ECM		[78]
Ag/O-Ti_3_C_2_T_x_/SiO_2_/Si	Bipolar switching	3.75(Dark)/2.75(UV light)	-9.25	10^4^	/	/	10.7 × 10^–5^(Dark)/10.7 × 10^–5^(UV light)	/	ECM	/	[53]
ITO/Ti_3_C_2_T_x_/EGaIn	Bipolar switching	1	−2.5	10^3^	10^4^	5 × 10^2^	1.72 × 10^–14^	1 × 10^–4^	ECM	Pavlov’s dog	[54]
Ag/ZIF-8:Ti_3_C_2_T_x_/FTO	Bipolar switching	2.5	−2.5	/	/	/	/	/	ECM	EPSC	[80]
PET/ITO/f-Ti_3_C_2_T_x_/Ag	Bipolar switching	0.72	−1	10^2^	10^6^	10^4^	0.24 × 10^–9^	/	Charge trapping	PPF/LTP/LTD/STDP Hebbian/anti-Hebbian	[116]
Ag/Ti_3_C_2_T_x_/SiO2/Pt	Bipolar switching	0.2	−0.2	10^4^	/	/	/	/	ECM	LTP/LTD	[117]
Ag/Ti_3_C_2_T_x_-PVA/ITO/PET	Bipolar switching	2	−2	10	10^4^	5 × 10^1^	/	/	Charge trapping	/	[118]
Au/Ti_3_C_2_T_x_/Y:HfO_2_/FTO	Bipolar switching	−3.7	3.5	10^3^	3 × 10^3^	/	/	/	VCM	/	[56]
Cu/Ti_3_C_2_T_x_/Cu	Bipolar switching	0.48	0.29	/	/	/		0.5 × 10^–7^	/	/	[119]
Ag/MoO_3_/OL/ZnO/FTCE/PEN	Bipolar switching	0.6	−0.4	10^3^	10^4^	4 × 10^3^		0.54 × 10^–5^	Charge trapping	LTP/LTD/STDP	[92]

GeO_x_-coated Ti_3_C_2_T_x_-based synaptic TFTs	Unipolar switching	/	/	/	/	/	10 × 10^–15^	/	Charge trapping	IPSC/EPSC/Visual perception	[40]
rGO/FE-Ti_3_C_2_T_x_/nFE-Ti_3_C_2_T_x_/rGO	Bipolar switching	−1.6	2.8	10^3^	4 × 10^3^	10^3^		1.6 × 10^–7^	VCM	/	[120]
Al/Ti_3_C_2_T_x_@MAPbI_3_/Al	Bipolar switching	2.22	−0.42	10^6^	10^4^	/	/	2.22 × 10^–8^	Charge trapping	/	[70]
Pd/Ti_3_C_2_T_x_@Cu-TCPP@PVA/Pt	Bipolar switching	1.35	−1.5	10^2^	/	/	/	2 × 10^–4^	VCM	LTP/LTD	[121]
rGO/HT-Ti_3_C_2_T_x_/Pd	Bipolar switching	2.5	−2.3	/	/	/	/	/	Charge trapping	/	[122]
Ti_3_C_2_T_x_/BFO/h-Ti_3_C_2_T_x_/Ti_3_C_2_T_x_	Bipolar switching	3	−1	10^2^	10^4^	10^3^	/	/	Electron tunneling	/	[64]
Ag/cellulose-Ti_3_C_2_T_x_ composite hydrogel/ITO	Threshold switching	± (0.5–1 V)	± (0.5–1 V)	/	/	/	/	/	ECM/VCM	LTP/LTD/STDP	[57]
Ag/TiO_x_/Ti_3_C_2_T_x_/Au	Bipolar switching	2.3	−2.5	10^2^	10^3^	/	/	2.3 × 10^–3^	ECM	LTP/LTD/Tactile perception	[31]
Fibrous: Ag/Ti_3_C_2_T_x_/Pt	Threshold switching	0.53/−0.55	0.1/−0.07	10^2^	5 × 10^2^	/	/	17 × 10^–9^	ECM	STM/LTM/Tactile perception	[27]
Au/PDVT−10/Ti_3_C_2_T_x_/PVN/SiO_2_/Si	Unipolar switching	/	/	/	/	/	/	/	/	IPSC/EPSC/PPF	[123]

Au/Ti_3_C_2_T_x_-PVP/rGO	Bipolar switching	0.9	−1.8	10	8 × 10^3^ s	/	/	0.9 × 10^–4^	Charge trapping	/	[124]
Ag/CN-Ti_3_C_2_T_x_/Pt	Bipolar switching	1.28	−1.36	10	/	/	/	1.28 × 10^–6^	ECM	Neurobiochemical responding/Damaged neuron	[30]
Au/OSC/MXP/Si/SiO_2_	Unipolar switching	/	/	10^5^	10^3^	10^5^	/	/	Charge trapping	EPSC/IPSC/PPF/LTP/LTD	[88]
Au/TAPA-Ti_3_C_2_T_x_/Au	Unipolar switching	/	/	/	250	10^8^	7.8 × 10^–12^	/	Charge trapping	/	[125]
m-CNTs/Al_2_O_3_/Ti_3_C_2_T_x_/HPI/m-CNTs/PI	Unipolar switching	/	/	1.52 × 10^3^	10^3^	/	/	/	Charge trapping	EPSC/IPSC/PPD/LTP/LTD/Pavlov’s dog	[91]
Au/Graphene/Ti_3_C_2_T_x_/TiO_2_/SiO_2_/Si	Unipolar switching	/	/	/	10^5^	/	/		Charge trapping	LTP/LTD	[126]
Al/Ti_3_C_2_T_x_/Pt/SiO_2_/Si	Bipolar switching	5.8	−5.5	3 × 10^4^	4 × 10^6^	10^4^	/	5.8 × 10^–4^	Charge trapping	/	[25]
Au/LPE/Ti_3_C_2_T_x_/Si	Unipolar switching	/	/	/	30		6.48 × 10^–12^	6.4 × 10^–12^	Charge trapping	EPSC/PPF/SRDP/SDDP/Pavlov’s dog	[17]
Cu/Ti_3_C_2_T_x_/PZT/Pt	Bipolar switching	1.2	−0.8	10^4^	3 × 10^3^	10^2^		1.2 × 10^–2^	ECM	PPF/STDP	[127]
Cu/Ti_3_C_2_T_x_/Au	Unipolar switching	/	/	/	/	/	/	/	ECM	LTP/LTD	[41]

Ag/Ti_3_C_2_T_x_/FTO	Bipolar switching	0.58	−0.55	3.6	10^3^	/	/	≈4.5 × 10^–2^	ECM/VCM	Visual perception	[128]
Ag/Ti_3_C_2_T_x_/C/PET	Bipolar switching	1.7	−1.5	3	/	/	3.8 × 10^–11^	/	ECM	EPSC/Tactile perception/Operant conditioning flex	[26]
Au/PDVT−10:Ti_3_C_2_T_x_/SiO_2_/Si	Unipolar switching	/	/	/	/	/	/	/	Charge trapping	EPSC/IPSC	[129]
ITO/PVA/Ti_3_C_2_T_x_/Ag	Unipolar switching	4	/	10^4^	/	/	/	/	ECM	LTP/LTD/EPSC/IPSC/STM/LTM/PPF/PPD	[23]
Pt/Ti_3_C_2_T_x_-TiO_2_/SiO_2_/Si	Unipolar switching	/	/	/	/	/	/	/	Charge trapping	STM/LTM/Visual perception/respiratory dynamics	[101]
Al/InO_x_/TiO_2_/Ti_3_C_2_T_x_/SiO_2_/n + + Si	Unipolar switching	/	/	/	/	/	0.22 × 10^–15^	/	Charge trapping	EPSC/Visual perception	[130]
ITO/PVA:Ti_3_C_2_T_x_-Ag NPs/Ag	Bipolar switching	4.37	−4.8	10^4^	10^2^	、		4.37 × 10^–9^	ECM	Visual perception	[107]
Pt/Ti_3_C_2_T_x_/Pt	Bipolar switching	5.03	−5.5	5.62 × 10^3^	10^4^	10^3^	/	5.03 × 10^–7^	VCM	LTP/LTD/PPF/PPD	[131]
Ag/Ti_3_C_2_T_x_/C/PET	Unipolar switching	0.75	−0.65	10^2^	/	/	/	2.1 × 10^–7^	ECM	Tactile perception	[94]

ITO/Ti_3_C_2_T_x_-ZnO/Al/PDMS	Unipolar switching	−0.31	1.3	10^4^	10^4^	4 × 10^2^	/	/	VCM	/	[42]
Au/Oxidized Ti_3_C_2_T_x_/SiO_2_/Si	Unipolar switching	/	/	10^6^	10^4^	3 × 10^2^	/	/	Charge trapping	LTP/LTD/PPF	[37]
Au/Ti_3_C_2_T_x_/VP	Bipolar switching	2	−2	/	/	/	/	/	Charge trapping	EPSC/PPF/STP/LTP/Visual perception/Olfaction perception	[132]
Al/Ti3C2Tx:Ag/Pt	Bipolar switching	0.2	0.2	10^3^	10^5^	10^6^	0.35 × 10^–12^	/	ECM	PTP/PPF/EPSC	[32]
Ag/Ti_3_C_2_T_x_/GST/Pt	Threshold switching	0.38	0.05	10^3^	10^4^	10^3^	/	1 × 10^–10^	ECM	/	[133]
Au/Ti_3_C_2_T_x_-TiO_2_/SiO_2_/Si	Unipolar switching	/	/	/	/	/	/	/	Charge trapping	STM/LTM/PPF/EPSC	[134]
TiN/Cu/Ti_3_C_2_T_x_/SiO_2_/TiN	Bipolar switching	1.8	−2	5	/	/	/	3.6 × 10^–4^	ECM	/	[135]
Fibrous/Ag/Ti_3_C_2_T_x_/MoS_2_/Pt	Threshold switching	0.75/−0.75	0.25/−0.3	10^2^	/	/	/	3.3 × 10^–10^	ECM	Immune compensation	[29]
Cu/Ti_3_C_2_T_x_/SiO_2_/W	Bipolar switching	1.75	−1	10^5^	10^3^	/	/	/	ECM	/	[136]
Cu/Ti_3_C_2_T_x_/BFO/Pt	Bipolar switching	0.6	−0.4	10^3^	1.2 × 10^3^	10^2^	/	/	Electron tunneling	LTP/LTD/PPF/STDP	[137]

Ag/Ti_3_C_2_T_x_/ITO	Bipolar switching	1	−1	/	/	/	5 × 10^–13^	7 × 10^–6^	ECM	Circadian Learnability	[105]
TiN/Cu/Ti_3_C_2_T_x_/SiO_2_/TiN	Bipolar switching	1.8	−1.8	/	/	/	/	/	ECM	/	[138]
Au/Ti_3_C_2_T_x_/TiO_2_/Au	Unipolar switching	/	/	/	/	/	/	/	/	PPF/Discomfort perception	[28]
Ag/BiFeO_3_/Ti_3_C_2_T_x_/FTO	Bipolar switching	3.5	−3.1	4	/	/			Electron tunneling	/	[65]
PET/ITO/p-Ti_3_C_2_T_x_/Ag	Unipolar switching	4.1	−5.9	10^3^	10^6^	10^4^	1.28 × 10^–7^	/	ECM	STDP/LTP/LTD	[116]
Au/P0FDIID:N2200/Ti_3_C_2_T_x_/Al_2_O_3_/Au/SiO_2_/Si	Bipolar switching	/	/	/	/	/	/	/	Charge trapping	Photoelectric sensor/Satellite remote sensing image recognition	[69]
Au/Ti_3_C_2_T_x_/Au	Bipolar switching	/	/	/	/	/	/	/	Charge trapping	STP/STD/LTP/LTD/STM/LTM/associative learning behaviors	[102]
Au/DPA/Ti_3_C_2_T_x_/Au	Unipolar switching	/	/	/	/	/	/	/	Charge trapping	EPSC/PPD	[139]

**Fig. 22:**
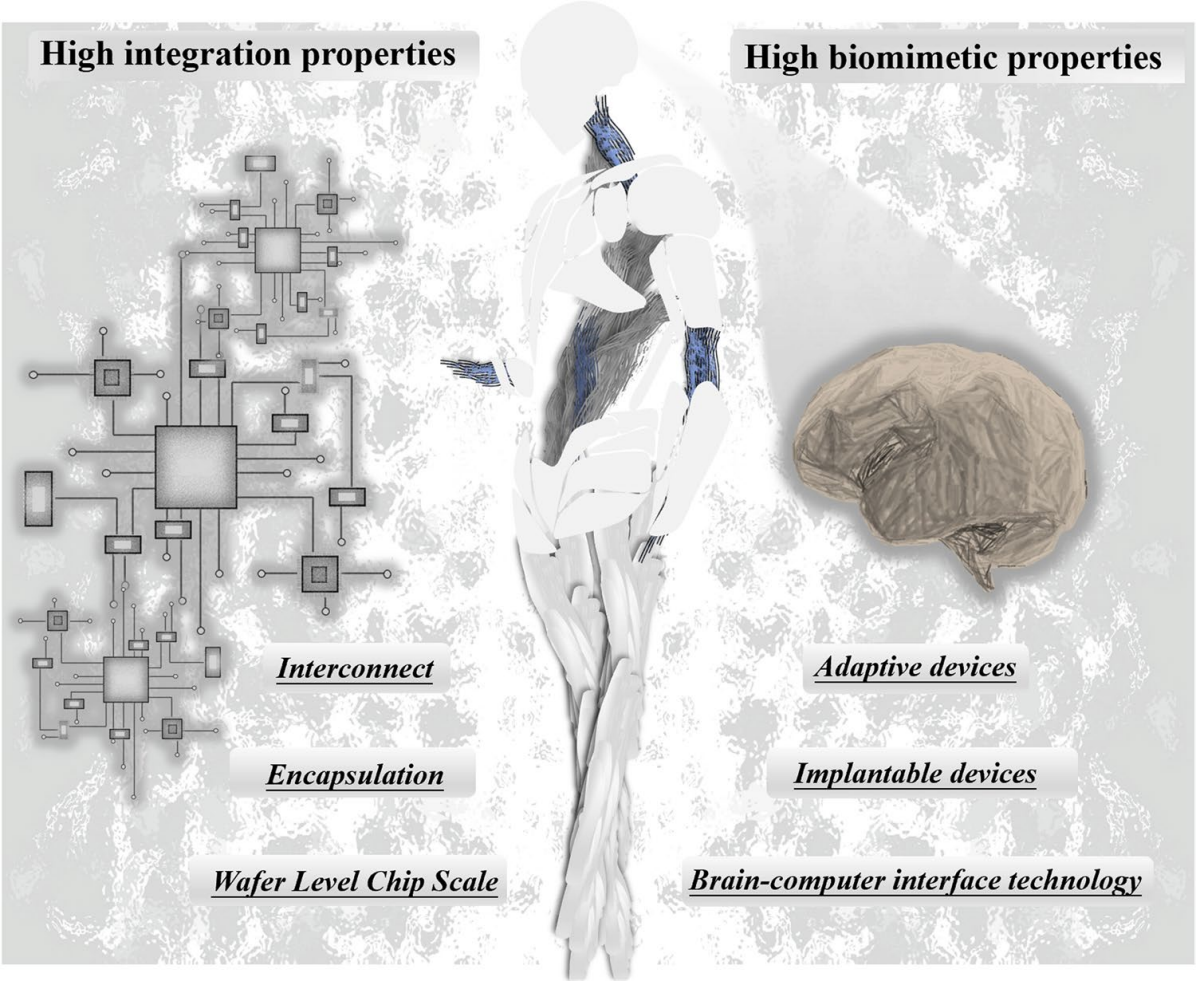
Future work trend for MXene-Ti_3_C_2_T_x_-based neuromorphic chip

However, although MXene-Ti_3_C_2_T_x_-based neuromorphic devices have made significant progress, they still face many challenges, including material stability, large-scale integration technology, and optimization of energy efficiency ratio, which urgently need further in-depth research and solutions. The current results show that neuromorphic devices based on MXene-Ti_3_C_2_T_x_ materials are advancing in two areas, which are high integration and high biomimetic properties (Fig. 22). To advance the practicalization of MXene-Ti_3_C_2_T_x_-based neuromorphic devices, the enhancement of large-scale integration capability and biocompatibility still face challenges, and broadening their application scope hinges crucially on these breakthroughs. Therefore, future research should focus on solving these core challenges to accelerate the practical application of MXene-Ti_3_C_2_T_x_-based neuromorphic devices.

(1) High integration properties: Currently, research on artificial synapse-based neuromorphic sensitive devices primarily focuses on device-level preparation and proof-of-concept, which initially meets the basic requirements of neuromorphic computing. Compared to CMOS technology that relies on standardized lithography processes, MXene device integration must overcome challenges in nanoscale patterning uniformity and inter-device crosstalk suppression issues less critical in conventional digital circuits but paramount for analog neuromorphic arrays. To address the above challenges, a layered nanofabrication strategy can be adopted. This strategy involves enhancing the precision of directional alignment of MXene during the substrate pre-patterning stage. Furthermore, it entails developing neuromorphic arrays based on 3D vertically integrated architectures, in combination with techniques such as atomic layer deposition (ALD). Additionally, electromagnetic shielding layers should be introduced to reduce interference.

However, how to efficiently synthesize functional materials at the wafer-level scale in order to achieve large-scale production and integration of such devices remains a research gap. Although studies have attempted to explore device integration applications by constructing bionic neuromorphic systems and have verified their potential for preparing neuromorphic devices, the issues of process control difficulty, implementation of wafer-level functionalization, and film quality control still need to be addressed in depth. In addition, large-scale integration needs to ensure that there is no interference between the memory cells. Therefore, the isolation and interconnection mechanism of the device becomes a key issue. Meanwhile, in the research of flexible and fibrous neuromorphic devices, how to avoid mechanical damage during motion under the layout of large-area arraying is also an important issue that needs to be solved.

(2) High biomimetic properties: Biocompatibility refers to the interaction of bionic materials with organisms without triggering immune or toxic reactions. Currently, biomimetic proximal computing research focuses on two main directions. First, it aims to mimic the biological afferent nervous system and design biomimetic systems to reproduce biological movement patterns. The second is to develop an autonomic nervous system suitable for organisms and realize the replacement of native ANS by implantation in the body. To enhance the biocompatibility of the biomimetic ANS, it is crucial to start from the material selection and preparation process. Using a biomaterial manufacturing process, MXene-based biomimetic conductive polymers can be produced, featuring degradation products that transition from non-fully bioidentical to fully biocompatible. This involves selecting non-toxic materials and adopting environmentally friendly processes to ensure that the preparation process is harmless. Alternatively, encapsulating the ANS through organic and polymer materials with high biocompatibility can reduce rejection reactions after implantation. In particular, with the rapid development of human–computer interaction and brain–computer interaction technologies, higher requirements have been imposed on biomimetic ANS. Specifically, accurate and efficient bioelectrical signal interaction interfaces need to be constructed, while ensuring a high degree of comfort in contact with the human epidermis, to achieve true bio-integration.

In conclusion, the outstanding performance of 2D MXene-Ti_3_C_2_T_x_ materials in neuromorphic computing not only highlights their far-reaching impact in the optoelectronic and semiconductor fields, but also opens up a brand-new path for the innovative development of memristor-based neuromorphic chips. By integrating sensing, memory, and computation in a single material platform, MXene-Ti_3_C_2_T_x_ overcomes the functional fragmentation of CMOS technology, paving the way for intelligence systems with high efficiency. Although research on MXene-Ti_3_C_2_T_x_-based neuromorphic chips is still in its infancy, its successful application in the fields of energy storage, sensor technology, and flexible electronic devices undoubtedly lays the foundation for advancing the development of the new generation of information technology. This will steadily lead us into a new era of more intelligent and efficient computing.
